# Development of Novel Neratinib and Docetaxel Core-Loaded and Trastuzumab Surface-Conjugated Nanoparticle for Treatment of HER-2 Positive Breast Cancer

**DOI:** 10.3390/pharmaceutics17101265

**Published:** 2025-09-26

**Authors:** Victor Ejigah, Gantumur Battogtokh, Bharathi Mandala, Emmanuel O. Akala

**Affiliations:** Center for Drug Research and Development, Department of Pharmaceutical Sciences, College of Pharmacy, Howard University, Washington, DC 20059, USA

**Keywords:** active targeting, nanoparticles, HER2-positive breast cancer, antibody-nanoparticle conjugate, synergistic effect, synchronized delivery

## Abstract

**Background/Objectives**: This study developed a targeted drug delivery nanoplatform for treating HER2-positive breast cancer. The nanoplatform encapsulated two hydrophobic anticancer agents, neratinib (NTB) and docetaxel (DTX), within nanoparticles (DTX+NTB−NP) functionalized for conjugation to trastuzumab to form trastuzumab-tagged nanoparticles (TRZ−NP). Trastuzumab is a HER2-specific monoclo-nal antibody that binds to HER2 receptors, blocking signal transduction and inducing an-tibody-dependent cellular cytotoxicity (ADCC). Upon receptor-mediated endocytosis, neratinib inhibits cytosolic HER2 signaling, while docetaxel disrupts mitotic cell division, collectively leading to tumor cell death. **Methods**: Nanoparticles were fabricated by the nanoprecipitation technique, followed by surface modification with a crosslinker and a targeting moiety. DTX+NTB−NP, TRZ−NP, and singly loaded nanoparticles (NTB−NP and DTX−NP) were characterized and their effects evaluated in HER2-positive cancer cell line and xenograft model. **Results**: In vitro antiproliferation assay in SKBR-3 cell line re-veals a dose and time-dependent cytotoxicity. There was no significant difference in cyto-toxicity observed between DTX+NTB−NP and its free form (DTX+NTB) [p = 0.9172], and between TRZ−NP and its free form (TRZ+DTX+NTB) [*p* = 0.6750]. However, TRZ−NP, at half the concentration of the singly loaded nanoparticles, significantly reduced the viabil-ity of SKBR-3 cells compared to pure trastuzumab (TRZ) [*p* < 0.001], NTB−NP [*p* = 0.0019], and DTX−NP [*p* = 0.0002]. In vivo evaluation in female athymic nude mice showed sig-nificant log relative tumor volume (%) reduction in groups treated with TRZ−NP and DTX+NTB−NP compared to PBS (phosphate-buffered saline) controls (*p* ≤ 0.001 and *p* ≤ 0.001), respectively. Notably, TRZ−NP demonstrated a statistically significant regression in the log relative tumor volume (%) compared to DTX+NTB−NP (*p* = 0.001). **Conclusions**: These findings underscore the therapeutic potential and suitability of these nanoplatforms for the precise and controlled targeting of HER2-positive tumors. This study is the first to synchronize the delivery of multiple agents-docetaxel, neratinib, and trastuzumab-within a nanoparticle system for treating HER2-positive tumors, offering a promising strategy to enhance treatment outcomes for HER2 positive breast cancer patients.

## 1. Introduction

The 2019 report of the incidence and mortality of cancers by the National Cancer Institute (NCI) revealed that breast cancer was the second leading cause of cancer deaths in the United States of America [1]. Progress in breast cancer therapy has changed that statistic, as recent data show that breast cancer is now the fourth most common cause of cancer death in the USA [2]. However, the incidence of breast cancer has risen considerably, making it the most commonly diagnosed cancer in the United States [2]. These statistics emphasize the public health importance of breast cancer.

Six molecular classifications of breast cancer have been identified through microarray gene expression analysis and unbiased hierarchical clustering. These are basal-like, human epidermal growth factor receptor 2 positive (ErbB2+/HER2+), normal breast-like, claudin low, luminal subtype A, and luminal Subtype B [3]. Prominent among these molecular subtypes is the HER2-positive breast cancer that is associated with overexpression of the human epidermal growth factor, driven by the amplification of the HER2/neu gene. This overexpression is a key marker of aggressive tumorigenesis and metastasis [4]. Nearly 20 to 30% of human breast cancers exhibit HER2+ receptor overexpression [5].

HER2-positive breast cancer has one of the poorest prognoses among breast cancer subtypes, surpassed only by triple-negative breast cancer [6]. HER2+ receptor overexpression is also observed in about 20% of gastroesophageal junction and gastric cancers, both associated with similarly poor prognosis [7,8]. HER2 is a type I transmembrane glycoprotein receptor tyrosine kinase involved in regulating cell proliferation, survival, and apoptosis [9]. HER2 belongs to the epithelial growth factor receptor (EGFR) family, which also includes EGFR (HER-1), HER-3, and HER-4. HER2 can form homodimers or heterodimers with other members of the EGFR family, triggering downstream signaling pathways through tyrosine phosphorylation [6,10]. These pathways modulate various cellular functions critical to cancer progression [6,10].

A major challenge in treating HER2-positive breast cancer is resistance to therapy [11,12], which arises from crosstalk between various cellular proliferation pathways and from the effect of efflux pumps [13]. Additionally, the cardiotoxicity and gastrointestinal side effects of pertuzumab—a first-line drug in HER2+ breast cancer treatment—limits long-term use [14,15]. Other issues include the toxicity of non-specific cytotoxic drugs and the irregular, non-synchronized pharmacokinetic profiles of these treatments, which further complicate effective therapy [16,17].

To find a ‘magic bullet’ that targets cancer cells while avoiding healthy cells, several strategies have been investigated, including the use of nanoparticles (NPs) to deliver multiple drug molecules per biorecognition event and prevent nonspecific release of their payload. Although this strategy is in its infancy relatively speaking, advancements such as antibody-drug conjugates [e.g., ado-trastuzumab emtansine (T-DM1), trastuzumab deruxtecan (T-DXd)], and abraxane, already approved by FDA, highlight the significant potential of this approach [18]. However, ADCs are prone to resistance in situations of reduced expression of HER2 receptors [11], upregulation of alternative receptor tyrosine kinases, increased expression of efflux transporters [19], impaired lysosomal release of lysine MCC-DM1 (active DM1 metabolite), and enhanced recycling of HER2-T-DM1 complexes, which is associated with reduced DM1 intracellular release [20].

Attempt to develop ADCs with a bystander effect has led to a couple of withdrawals due to debilitating side effects [21].

This project investigates the safe and precise delivery of a stealth targeted nanocarrier system of trastuzumab (a monoclonal antibody that binds HER2 receptor outside the tumor cells) conjugated to nanoparticles encapsulating neratinib (a small molecule tyrosine kinase inhibitor that binds HER2 receptor inside the tumor cells) and docetaxel (a cytotoxic agent) for synchronized delivery and synergistic treatment of HER2-positive breast cancer.

The proposed multifunctional nanotechnology drug delivery system has the capability to:

- Minimize non-specific drug biodistribution to reduce chemotherapy-associated toxicity.
- Enhance synergy through three agents with orthogonal mechanisms of action, effectively blunting downstream signaling.
- Synchronize the pharmacokinetics (simultaneous delivery at the biophase) of all three agents to mitigate upregulation of alternate signaling pathways arising from differences in drug absorption and onset of action.
- Improve treatment adherence by reducing dosing frequency.
- Overcome drug resistance by (a) preventing nanoparticle removal via p-glycoprotein efflux pumps and (b) enabling multi-drug delivery per molecular recognition event using targeted nanoparticles, thus preventing cellular crosstalk.
- Establish a safe, effective, and precise nanocarrier-based drug delivery approach, with potential applications in broader oncological and drug delivery challenges.

## 2. Materials and Methods

### 2.1. Materials

PLGA-PEG-COOH, PLGA-PEG-N3 (PolicyTech, Lafayette IN, USA). Methanol HPLC grade, sodium citrate, acetic acid, acetonitrile, docetaxel, neratinib, and acetone (HPLC grade > 99%) were all obtained from Fisher scientific. EDC.HCL [(1-(3dimethyl amino propyl)-3-1 ethyl carbodiimide hydrochloride) (ThermoFisher Scientific, Waltham, MA, USA)], NaIO_4_ (sodium per iodate), Purpald reagent, NaCNBH_3_ (sodium cyanoborohydride), sodium acetate and 30kDa (MWCO) membrane were obtained from Sigma Aldrich, St. Louis, MO, USA. Other materials used include the following: di-H2O, acetonitrile and methanol (HPLC grade, and acetic acid (Fisher Scientific, Pittsburgh, PA, USA), trifluoroacetic acid > 99% (Chem-Implex), trastuzumab (Genentech, San Francisco, CA, USA) Tween 80 (ICI Inc., Washington, DC, USA), dialysis membrane (12–14 KDa MWCO (Spectra/Por^®^ CE, Spectrum Labs, Rancho Dominguez, CA, USA), Matrigel (Corning^®^, Corning, NY, USA), BD alcohol swabs, female athymic nude mice (strain #490, Charles River Laboratories, Wilmington, MA, USA).

### 2.2. Instruments

Phenom^®^ Desktop SEM Pure Scanner, (ThermoFisher Scientific), Eppendorf centrifuge^®^ (Ocala, FL, USA) Daigger Vortex-Genie 2, magnetic stirring plate (Isotemp), magnetic stirring bar, Dynamic Light Scattering particle size analyzer/zetasizer (Brookhaven 90 plus^®^, Nashua, NH, USA), 200 kV FEI Talos F200X TEM, Fourier-Transformed Infra-Red (Perkin Elmer Spectrum 100, Springfield, IL, USA) spectrometer, UV Spectrometer (Shimadzu, Columbia, MD, USA), Mass Spectroscopy (LC-MS, Agilent, Santa Clara, CA, USA) Matrix assisted Laser Desorption Ionization-Time of Flight-Mass Spectroscopy (Bruker Autoflex Speed MALDI-TOF MS, Billerica, MA, USA), lyophilizer (Labconco^®^, Kansas City, MO, USA). GPC (Gel permeation chromatography) mounted with a Phenogel column (5 μm 104, 103, 500 Å, Dimensions: 300 mm × 7.8 mm); data were processed with the versatile Empower^®^ software (Waters Corp. Version 3.8.1, Milford, MA, USA), Reverse-Phase High-performance Liquid Chromatography (R-HPLC, Hewlett Packard-Agilent Series 1100, Santa Clara, CA, USA) equipped with a column (Zorbax 300SB-C18, 5 um, 4.6 mm × 250 mm, Santa Clara, CA, USA), autosampler, quaternary pump, photodiode array detector, and column thermostat. Data analysis and processing were performed with the proprietary software Chemstation (Agilent Technologies, Santa Clara, CA, USA). The binding study was performed with Cytoflex^®^ flow cytometer (Beckman Coulter Inc., Brea, CA, USA) and data analyzed with Cytexpert^®^. Cytotoxicity study was performed and analyzed with CLARIOstar Plus Microplate Reader (BMG Tech, Norristown, PA, USA). Animals were weighed on a scale (Sartorious LE5201, Cambridge, MA, USA), restrained with a mouse tail illuminator (Brain Tree Science Inc., Brain Tree, MA, USA) and tumor volume measured with a digital caliper (150 mm, World Precision International, Sarasota, FL, USA).

### 2.3. Methods

#### 2.3.1. Formulation of Dual-Loaded Nanoparticles (DTX-NTB-NP)

Nanoparticles were fabricated by the modification of a previously reported nanoprecipitation method [22]. Briefly, PLGA-PEG-COOH and PLGA-PEG-N3 in a ratio of 18:1 were co-dissolved with neratinib + docetaxel in acetone, followed by dropwise addition to a mixture of water and acetone while stirring at 1200 rpm. The fabricated nanoparticles were centrifuged and washed with diH2O before lyophilization (Labconco^®^, Kansas City, MO, USA) and characterization. All drug-loaded and blank nanoparticles were fabricated using the same procedure.

#### 2.3.2. Conjugation of Monoclonal Antibody (Trastuzumab) to Drug-Loaded Nanoparticles

The coupling of trastuzumab (TRZ) to drug-loaded nanoparticles followed a previous method used in our laboratory [23]. First, TRZ was purified by six rounds of washing and filtration with acetate buffer (0.1 M, pH 5.5) using Amicon filters (30 kDa MWCO) by centrifugation in an Eppendorf^®^ 5417R centrifuge (Framingham, MA, USA). The process was conducted at 4 °C using 10,000× *g* RCF for 10 min. The purified TRZ was then quantified at 280 nm using a Shimadzu UV-2401PC spectrophotometer (Columbia, MD, USA).

#### 2.3.3. Oxidation of Trastuzumab (Ox-TRZ)

TRZ was oxidized with a 50–200 molar excess of NaIO_4_ (sodium periodate) by incubating it in the dark at room temperature for 30 min under gentle agitation. The resulting Ox-TRZ, containing reactive aldehydes, was purified using a 0.1 M sodium acetate buffer (pH 5.5) and an Amicon 30 kDa MWCO spin filter to remove excess NaIO_4_ [23,24]. A sample of the Ox-TRZ was analyzed for the presence of aldehyde groups using Purpald reagent (Sigma Aldrich, USA, St. Louis, MO USA). The presence of aldehyde groups was further verified by UV analysis of a sample of Ox-TRZ at the formaldehyde absorption wavelength of 550 nm. The amount of trastuzumab recovered was quantified via an established formaldehyde calibration curve.

#### 2.3.4. Conjugation of Carbohydrazide to TRZ (TRZ-Carbohydrazide)

A molar excess of carbohydrazide was mixed with oxidized trastuzumab and allowed to react with gentle agitation for 2 h [23,25]. At the end of the reaction, the ensuing Schiff base was reduced with NaCNBH_3_ (sodium cyanoborohydride, Sigma Aldrich, St. Louis, MO, USA) under slight agitation at room temperature [26]. The conjugated TRZ-carbohydrazide was washed, and the amount recovered was quantified by UV analysis. UV analysis was also performed to detect the presence of an aliphatic hydrazone bond at a wavelength range of 290–400 nm [27,28] and the result compared with the UV absorption of oxidized trastuzumab and PBS under a similar condition.

#### 2.3.5. Conjugation of Trastuzumab-Carbohydrazide to Drug-Loaded Nanoparticles (TRZ-NP)

The carboxylic groups of DTX-NTB-NP were first activated with EDC.HCL (1-(3-dimethylamino propyl)-3-1 ethyl carbodiimide hydrochloride), washed and reacted with TRZ-carbohydrazide for 3 h [29]. At the end of 3 h, the TRZ−NP conjugate was washed, filtered, and stored at 4 °C.

#### 2.3.6. Confirmation of TRZ-NP Conjugation

The filtered TRZ−NP conjugate was centrifuged at 16,200× *g* RCF for 8 min as described previously [30]. The supernatant containing unconjugated trastuzumab-carbohydrazide was collected, and the pellet was resuspended in 1mL of 0.1 M PBS (pH 7.2). UV analysis of supernatant was performed to quantify the amount of unconjugated trastuzumab-carbohydrazide. The mass of trastuzumab conjugated to the drug-loaded nanoparticles was determined by subtracting the amount left in the supernatant from the amount used in conjugation.

#### 2.3.7. Confirmation of TRZ-NP Conjugation via BCA (Bicinchoninic Acid Assay)

According to the BCA protocol, bicinchoninic acid and copper II sulfate in the Pierce BCA kit (Thermofisher) were mixed in a ratio of 50:1 [31]. In this experiment, 10 mL of bicinchoninic acid was mixed with 0.2 mL of reagent copper II sulfate to form a 50:1 solution. 1 mg of TRZ−NP and DTX+NTB−NP were initially suspended in deionized water by vortexing. 0.1 mL of each sample plus 2 mL of assay mixture (1:20) were transferred into 1.5 mL microcentrifuge tubes and allowed to react at 37 °C for 30 min. This process was repeated for a sample of unconjugated trastuzumab. After 30 min, samples were scanned with a Shimadzu UV spectrophotometer in the wavelength range of 200–700 nm.

#### 2.3.8. Confirmation of TRZ-NP Conjugation by Analysis of Functional Groups

Fourier-transform infrared (FT-IR) spectroscopic analysis of TRZ−NP was conducted using a Perkin Elmer Spectrum 100 FT-IR spectrometer to monitor the presence and absence of key functional groups. This analysis included samples of TRZ−NP, TRZ, and TRZ-carbohydrazide.

#### 2.3.9. Characterization of Trastuzumab Conjugated Nanoparticles (TRZ−NP)

##### Particle Size and Zeta Potential

The average particle size, particle size distribution, and zeta potential of DTX+NTB−NP, singly loaded (DTX−NP and NTB−NP), and blank nanoparticles were determined using the dynamic light scattering technique with a Brookhaven 90 plus^®^ (Nashua, NH, USA) particle size analyzer at 25 °C. The measurements were conducted in triplicate as previously reported in our laboratory [32,33] and elsewhere [34].

##### Surface Morphology with SEM (Scanning Electron Microscopy)

Samples of drug-loaded nanoparticles were vacuum dried at 30 mmHg for 24 h in the presence of a hygroscopic agent (phosphorus pentoxide). The morphology of samples was determined with Phenom^®^ Desktop SEM Pure Scanner (ThermoFisher Scientific).

##### Internal Structure of the NPs (Transmission Electron Microscopy)

The structure of the nanoparticles was characterized via a previously published method [35] with a 200 kV FEI Talos F200X TEM system.

##### Characterization of Key Functional Groups in the Nanoparticles

Fourier-transform infrared (FT-IR) spectroscopic analysis of the fabricated nanoparticles was conducted using a Perkin Elmer Spectrum 100 FT-IR spectrometer to monitor the presence and absence of key functional groups. This analysis included samples of PLGA-PEG-COOH, DTX+NTB−NP, TRZ, and TRZ−NP.

#### 2.3.10. HPLC Method Development

Method development was performed with a Hewlett-Packard HPLC instrument (1100 series, Santa Clara, CA, USA) fitted with an Agilent autosampler (Series DE-23910399, Santa Clara, CA, USA), a column (Zorbax 300SB-C18, 5 um, 4.6 mm × 250 mm, Santa Clara, CA, USA), quaternary pump, photodiode array detector, column thermostat, and Chemstation analytical software (Agilent Technologies, Santa Clara, CA, USA) to simultaneously quantify neratinib and docetaxel.

#### 2.3.11. Encapsulation Efficiency, Drug Loading, and In Vitro Drug Release Profile

The encapsulation efficiency and drug loading were determined as previously published in our lab [33]; while the in vitro drug release from DTX+NTB−NP and TRZ−NP was performed by the dialysis method [36].

### 2.4. Cell Culture Assays

SKBR-3 cell line (American Type Culture Collection, ATCC, Manassas, VA, USA) was maintained in McCoy 5A medium supplemented with fetal bovine serum (FBS; 10% *v*/*v*), and penicillin/streptomycin [penicillin G (100 U/mL and streptomycin sulfate (100 ug/mL); 1% *v*/*v*]. Cells were cultured as a monolayer in T-75 flasks (Corning^®^) and maintained in a humidified incubator at 37 °C with supplemental CO_2_. Cells were regularly sub-cultured in new flasks as soon as they achieved 70–80% confluence. To ensure that TRZ retains the capacity to bind to HER2-positive receptors after conjugation to DTX-NTB-NP, a binding study assessment was performed via flow cytometry, while uptake of NPs was also evaluated to determine if NPs are localized within the cytoplasm. Cytotoxicity was assessed by cell proliferation studies using the CellTiter-Glo cell viability assay kit.

#### 2.4.1. Binding Study

Cells were preprocessed as previously described in our laboratory [23]. The cells were analyzed via flow cytometry using a Cytoflex^®^ flow cytometer (Beckman Coulter Inc., Brea, CA, USA.) at the blue laser wavelength (488 nm) that covers both propidium iodide (λ 488 nm) and FITC (λ 525/40 nm) excitation wavelengths. The acquired dataset was analyzed with Cytexpert^TM^ software 2.6 (Brea, CA, USA).

#### 2.4.2. Cell Uptake Study

Cell uptake of the NPs was analyzed using confocal laser scanning microscopy (CLSM 510) as previously published [33]. SKBR-3 cells were seeded onto 6-well plates at a density of 6 × 10^5^ cells per well in 2 mL of complete media and allowed to attach overnight in an incubator. 10 ug/mL of free Rhodamine 123 (Rhd, Invitrogen, Waltham, MA, USA), Rhd-loaded NPs, and Trastuzumab-Rhd-loaded NPs were added to each well and incubated with the cells for 6 h at 37 °C. To visualize internalization of nanoparticles, the cells were washed twice with cold PBS, fixed with 4% paraformaldehyde (2 mL) for 5 min, washed again with cold PBS once and further incubated with Hoechst R 33342 (5 μg/mL) (nucleus stain) and CellMask^TM^ deep red plasma membrane stain (4 μg/mL) for 1 min. Afterward, the slides were rinsed with PBS once. Finally, the slides were prepared with Fluoromount (Sigma, St. Louis, MO, USA), mounted on glass slides, and left to dry overnight at room temperature. Imaging was performed using a CLSM 510 (Carl Zeiss, GmbH, Oberkochen, Germany) equipped with a 60 × 1.3 NA Plan-Apochromat oil immersion objective and a multitrack configuration. Fluorescent signals from Hoechst R 33342, rhodamine-123-loaded nanoparticles, and CellMask^TM^ deep red plasma membrane stain were detected using BP 385–470 nm, BP 505–550 nm, and LP 650 nm filters after excitation with 364 nm, 488 nm, and 633 nm laser lines, respectively. Images (512 × 512 pixels) were captured with a line averaging of four using the Zeiss AIM software (ZEN analyzer).

#### 2.4.3. Cytotoxicity Assay

Cells were plated in 96-well microplates at a density of 5000 cells per 100 μL of culture medium and incubated for 24 h to promote attachment. Afterward, the medium was replaced with fresh culture medium containing varying concentrations (1 nM to 10,000 nM) of either the free-form of drugs (docetaxel, neratinib, trastuzumab, or their combinations) or drug-loaded nanoparticles. Control groups included wells treated with only culture medium, culture medium containing 0.01% DMSO, or blank polymeric nanoparticles.

### 2.5. Tumor Xenograft Model

All animal experiments were conducted according to an approved protocol (IACUC-PHARM-24-01: Approval Date: 12 March 2024, and Expiration Date: 11 March 2025) by the Institutional Animal Care and Use Committee (IACUC) at Howard University, Washington, DC, USA.

Tumor xenograft models were developed according to previously published methods [37,38]. Briefly, 5 × 10^6^ SKBR3 cells were suspended in 100 μL of PBS, mixed (1:1) with Matrigel (Corning^®^, Corning, NY, USA) and inoculated subcutaneously into the right flank of female athymic mice to generate xenograft tumors. Tumor formation was measured with fine electronic calipers (WPI^®^, Sarasota, FL, USA) every 3 days. Tumor volume was calculated by the formula:

Volume = (0.5 × Length × Width^2^) (1)

where Length = longest dimension, Width = measurement taken perpendicular to the length.

When the tumor volume reached ~100 mm^3^, the tumor-bearing mice were randomized for subsequent experiments.

#### 2.5.1. Toxicology Study/Maximum Tolerated Dose (MTD)

MTD experiments were performed according to a modification of a previous report [39]. Female athymic nude mice were randomly assigned to receive an escalating dose of TRZ−NP, DTX+NTB−NP, and a combined solution of TRZ+DTX+NTB at doses of 10 mg/kg, 20 mg/kg, and 30 mg/kg. The treatments were administered via tail vein injections twice, with a 3-day interval between doses. The animals were monitored for any changes in body weight, body posture, movement, social activity, and skin condition. In this study, MTD is defined as the highest tolerated dose that did not pose any life-threatening toxicity for the duration of the study. All the animals were euthanized after drug administration per approved IACUC protocol.

#### 2.5.2. Efficacy Study

The antitumor activity of TRZ−NP was evaluated by the modification of a previously reported method [40]. Once tumors were established, the mice were randomized and intravenously injected (tail vein injection) with either TRZ−NP, DTX+NTB−NP, or PBS, (30 mg/kg) once every 3 days. The tumor dimensions were measured with an electronic caliper every 2 days, and the tumor volumes were calculated using Equation (1). The mice were also observed for survival every 2 days.

### 2.6. Statistical Analysis

Particle size and zeta potential data for all nano-formulations and blank nanoparticles were analyzed using one-way ANOVA with GraphPad Prism software (version 10.2.1) and Origin Pro (version 2024b). Tukey’s post-hoc test was subsequently performed to identify specific differences among the formulations where the omnibus test was significant. For cell viability analysis, one-way ANOVA was used to compare the effect of the control group (medium alone), blank nanoparticles, and the secondary control group (medium + DMSO), utilizing the same software. Additionally, two-way ANOVA was employed to evaluate the in vitro cytotoxicity of different nano-formulations across various concentrations on SKBR3 cell lines. Tumor growth inhibition percent (TGI%) was computed to standardize treatment effectiveness over time. Tumor regression effects of TRZ−NP, DTX+NTB−NP, and PBS were also assessed using one or two-way ANOVA, with Tukey’s pairwise comparison test to detect specific differences. A significance level of 0.05 was used for all statistical analyses.

## 3. Results

### 3.1. Formulation and Characterization of Blank and Drug-Loaded Nanoparticles

#### 3.1.1. Particle Size Distribution

Single (neratinib or docetaxel), blank, and dual (neratinib/docetaxel) nanoparticles were fabricated via nanoprecipitation according to a previously published report [22] (Supplement Figure S1). TRZ−NP was synthesized via a series of conjugation reactions as described under methods. The hydrodynamic particle size of these formulations was measured in triplicate by the dynamic light scattering (DLS) technique. In this study, blank nanoparticles had an average size range of 87.4 nm to 95.8 nm, while the dual-loaded nanoparticles ranged from 156.3 nm to 166.4 nm. Single docetaxel- and neratinib-loaded nanoparticles demonstrated sizes that ranged from 129.7 nm to 145 nm. The particle size of the dual-loaded nanoparticles increased to a range of 261.3 nm to 296.6 nm after conjugation with the targeting moiety, trastuzumab.

**Figure 1:**
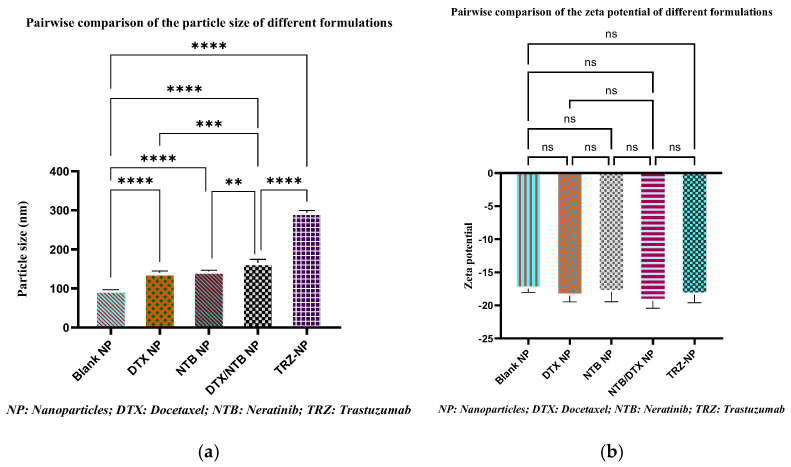
(**a**,**b**) Pairwise comparison of the particle sizes and zeta potential of different formulations. Significant differences in particle size between formulations are indicated by asterisks. The asterisks correspond to statistical significance levels, with more asterisks indicating stronger significance (** *p* = 0.01, *** *p* = 0.001, **** *p* < 0.0001, ns = non-significant).

A one-way ANOVA (analysis of variance) was performed to evaluate the influence of the addition of new molecules to the nanoparticles. Figure 1a presents a comparison of particle sizes across different nanoparticle formulations, including blank nanoparticles (Blank NP), docetaxel nanoparticles (DTX−NP), neratinib nanoparticles (NTB−NP), dual-loaded nanoparticles (DTX+NTB−NP), and trastuzumab-conjugated nanoparticles (TRZ−NP).

#### 3.1.2. Zeta Potential

The surface charge of the nanoparticles was evaluated with a zeta potential analyzer (Brookhaven 90plus, v2.18). The zeta potential of the various nano-formulations ranged from approximately −15 mV to −20 mV, indicating negatively charged surface characteristics across the board.

The impact of single and dual loading as well as the effect of trastuzumab conjugation on the zeta potential of the various nanoparticles was also assessed with a one-way ANOVA, and the result is presented in Figure 1b.

#### 3.1.3. Morphology and Structure of Nanoparticles

**Figure 2:**
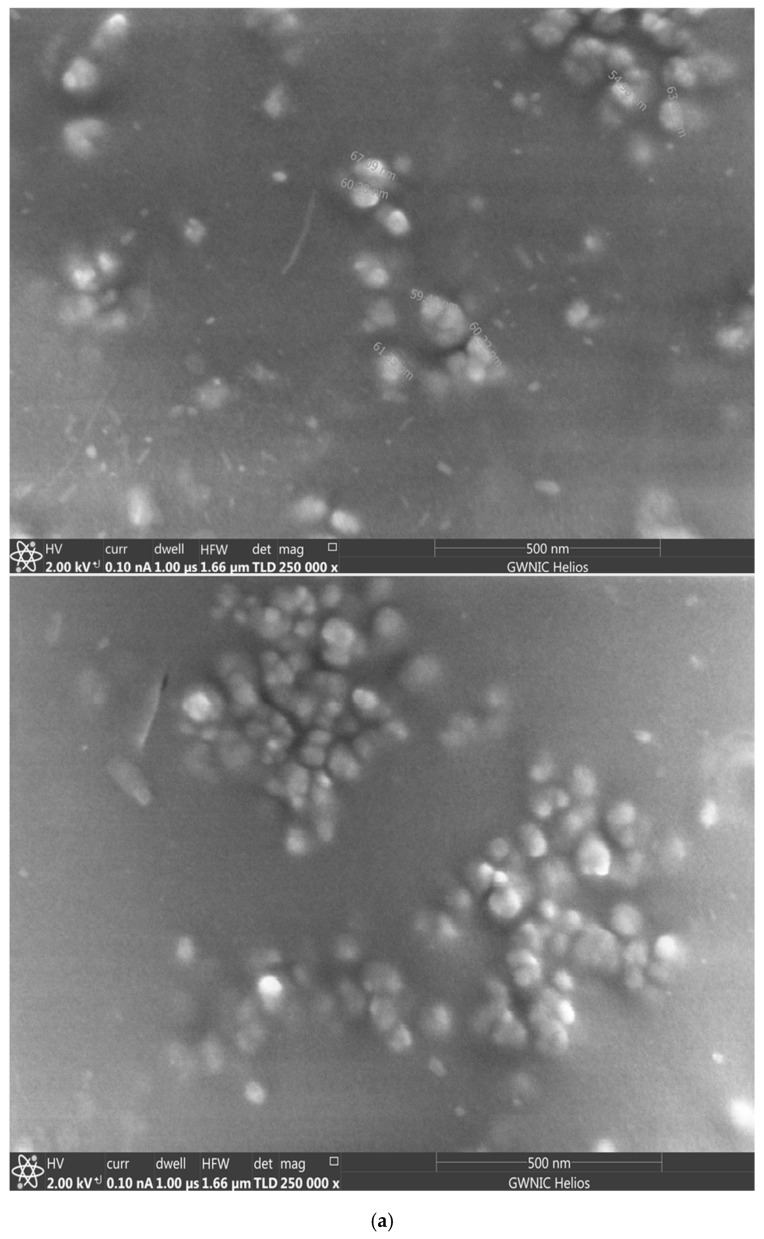

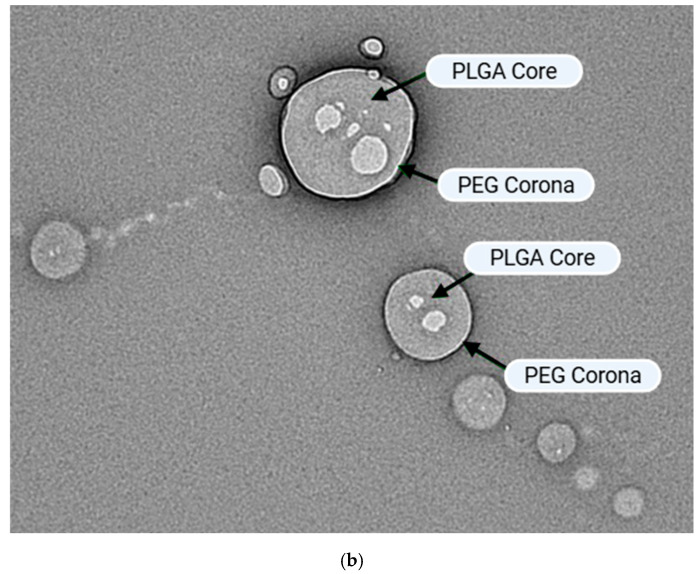
SEM and TEM images of drug-loaded nanoparticles showing clusters of spherical nanoparticles (**a**), and a cross-section of the nanoparticles showing hydrophobic core (PLGA) and hydrophilic PEG corona (**b**).

The successful formation of stealth nanoparticles was validated through visual confirmation using scanning electron microscopy (SEM) as shown in Figure 2a; while Figure 2b shows the structure of the stealth nanoparticles (PLGA core with a corona of PEG).

#### 3.1.4. Oxidation of Trastuzumab

The oxidation of carbohydrate residues in the FC region of trastuzumab was confirmed by the presence of aldehyde groups after Purpald’s assay (purple color). The reaction between formaldehyde and Purpald (4-amino-3-hydrazino-5-mercapto-1,2,4-triazole) occurs under alkaline conditions at room temperature. This method is non-destructive and offers superior sensitivity compared to other techniques, making it ideal for the assay of thermolabile biologics [41]. The presence of aldehyde groups was further verified by UV analysis at the formaldehyde absorption wavelength of 550 nm.

#### 3.1.5. Conjugation of TRZ to Carbohydrazide

The reaction between hydrazine and carbonyl groups often forms hydrazone bonds that are typically detected via UV or fluorescence analysis [27]. In this experiment, the hydrazone bond was verified with UV analysis at a wavelength range of 290–400 nm [27,28]. A comparison of the UV spectra of TRZ-carbohydrate with those of Ox-TRZ and PBS under similar conditions is shown in Supplement Figure S2. The stacked spectrometric analysis clearly shows the superior absorption of the hydrazone-containing sample (TRZ-carbohydrazide) compared to other samples tested at this absorption wavelength. The hydrazone bond formed was subsequently reduced to a stable hydrazine bond with NaCNBH_3_ [26]. The presence of the hydrazine bond was verified via FTIR, as shown in Supplement Figure S3.

#### 3.1.6. Conjugation of Trastuzumab-Carbohydrazide to Drug-Loaded Nanoparticles

**Figure 3:**
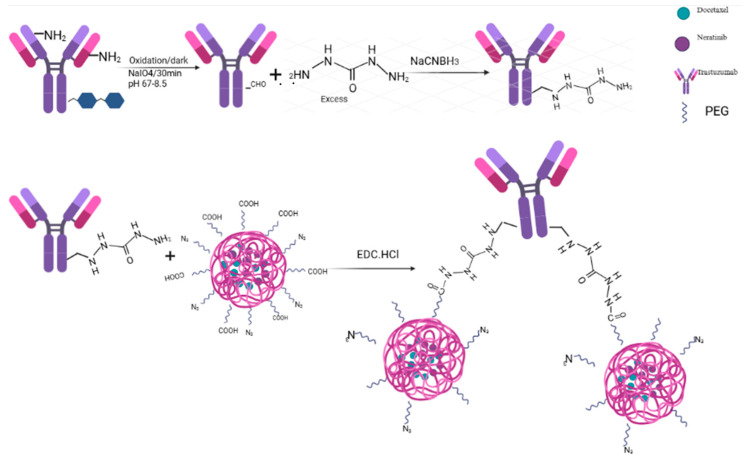
Illustration of the overall scheme in the development of trastuzumab conjugated drug-loaded nanoparticles.

The synthesis and conjugation process of trastuzumab to dual-loaded nanoparticles is illustrated in Figure 3. Previous studies from our laboratory have shown that a single molecule of trastuzumab can bind to two nanoparticle molecules through its available carbohydrate residues, which are oxidized for conjugation with a hydrazine crosslinking agent [23].

#### 3.1.7. Confirmation of TRZ−NP Conjugation by Centrifugation

Coupling of trastuzumab to nanoparticles was evaluated by centrifugation based on the assumption that only molecules of TRZ−carbohydrazide coupled to nanoparticles will sediment under force; while the uncoupled TRZ-carbohydrazide will remain suspended [30,42,43]. UV analysis of the supernatant was performed to quantify the amount of unconjugated TRZ-carbohydrazide. The mass of trastuzumab conjugated to drug-loaded nanoparticle was determined by subtracting the amount left in supernatant from the amount used in conjugation (5.4%).

(2) $$\%\;\mathrm{of}\;\mathrm{TRZ}\;\mathrm{conjugated}=\frac{\mathrm{Initial}\;\mathrm{amount}\;-\;\mathrm{amount}\;\mathrm{in}\;\mathrm{supernatant}\;\times 100}{\mathrm{Initial}\;\mathrm{amount}}$$

#### 3.1.8. Confirmation of TRZ−NP Conjugation by FTIR

The conjugation of TRZ to nanoparticles was also verified by FTIR. Samples of TRZ-carbohydrazide before conjugation to NP and the supernatant after conjugation to NP (which theoretically refers to the unconjugated TRZ−carbohydrazide) were compared as shown in Supplement Figure S4. The result presents similar spectra between the two samples.

#### 3.1.9. Confirmation of TRZ-NP Conjugation by Bicinchoninic Acid Assay (BCA)

**Figure 4:**
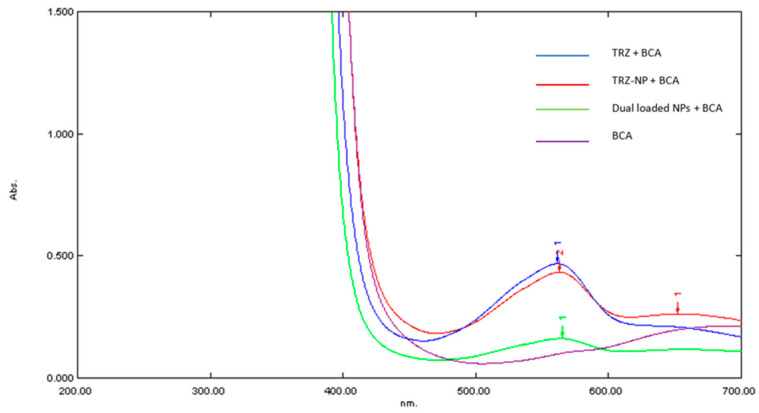
UV analysis of BCA-cupric ion complex in the presence of various samples. BCA: Bicinchoninic acid; TRZ−NP: Trastuzumab conjugated nanoparticles; NPs: Dual-loaded nanoparticles.

The conjugation of TRZ to nanoparticles was further validated with the BCA assay (Figure 4). The BCA assay operates on the principle that certain amino acids (tyrosine, tryptophan, cysteine, and cystine) can reduce copper (II) ions (Cu^2+^) to copper (I) ions (Cu^+^) under alkaline conditions [31]. The reduced copper (I) ions then react with two molecules of bicinchoninic acid (BCA) to form a purple-colored complex [44]. This complex exhibits strong absorbance at a wavelength of 562 nm, with the intensity directly proportional to the protein concentration in the sample [45].

### 3.2. HPLC Method Development for Quantifying Analytes

In this project, the gradient elution method was used as the appropriate method for the separation of neratinib and docetaxel. The final method employed a gradient strategy with 0.1% TFA (trifluoroacetic acid) in deionized water as mobile phase A and 100% acetonitrile as mobile phase B. Detection was performed at wavelengths of 230, 254, and 280 nm, with a flow rate of 1.2 mL/min, an injection volume of 20 µL, run time of 14 min and a column temperature of 37 °C.

### 3.3. Drug Release Profile (In Vitro), Drug-Loading and Encapsulation-Efficiency

The dialysis method was used to evaluate drug release from DTX+NTB−NP and TRZ−NP. To simulate the tumor microenvironment, this study utilized an acetate buffer (pH 5.5) for drug release investigations. The aliquots withdrawn at predetermined time points were analyzed to determine the in vitro drug release profile using the previously established HPLC method. Supplement Figure S5 shows the in vitro drug release isotherm of both docetaxel and neratinib from DTX+NTB−NP (a) and TRZ−NP (b). The release of both NTB and DTX in DTX+NTB−NP (Supplement Figure S5a) shows an initial rapid release within the first 24 h, followed by a gradual rate of release for an extended period. About 40–60% of the encapsulated drug was released at 24 h; while complete drug release was observed at approximately 150 and 200 h, respectively, for NTB and DTX, approaching 100% cumulative release, and suggesting that most of the drugs were released over this time frame. The drug release profile from TRZ−NP (Supplement Figure S5b) follows a similar pattern, though over a shorter period (120 h).

### 3.4. Binding Affinity of Antibody-Nanoparticle Conjugate

**Figure 5:**
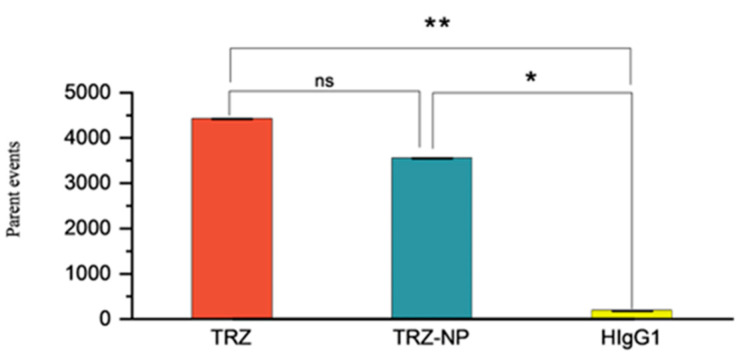
Pairwise comparison of FITC-derived percentage parent events after treatment with control (HIgG1, human immunoglobulin 1), TRZ (trastuzumab), and TRZ-NP (trastuzumab conjugated nanoparticles. The asterisks correspond to statistical significance levels, with more asterisks indicating stronger significance (* *p* = 0.05, ** *p* = 0.01, ns = not significant).

Data on percentage events were collected, analyzed, and presented as a plot of parent events vs. type of treatment (Figure 5). The data show that there is no significant difference in the binding affinity of TRZ and TRZ−NP in the FITC-positive gated cells (*p* = 0.505). Conversely, there was a significant difference in the binding affinity of TRZ vs. HIgG1 (*p* = 0.01) and TRZ−NP vs. HIgG1 (*p* = 0.04), largely due to the poor affinity of HIgG1 (control) for the biomarker (HER2 receptors). Data are further presented as overlay plots of histograms for cells treated with either TRZ, TRZ−NP, or HIgG1c (Supplement Figure S6).

### 3.5. Nanoparticle Uptake Studies

Cellular uptake was determined by CLSM. The results demonstrate the uptake of discrete particles at 6, 24 and 48 h. Supplement Figure S7 shows rhodamine-123-loaded (green color) nanoparticles surrounding the nucleus (blue) and bound by the plasma membrane (red).

### 3.6. Cytotoxic Effect of Docetaxel, Neratinib, and Trastuzumab on SKBR3 Cells

**Figure 6:**
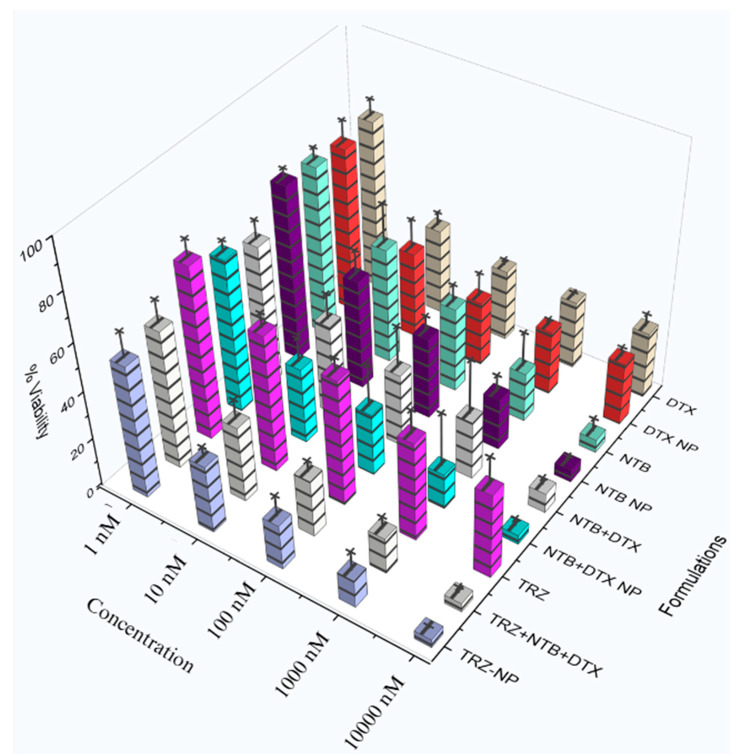
3D rendering of cell viability across varying concentrations at 120 h. DTX: Docetaxel solution, DTX−NP: Docetaxel nanoparticle, NTB: Neratinib solution, NTB−NP: Neratinib nanoparticles, NTB+DTX: solution, NTB+DTX−NP: Neratinib + docetaxel nanoparticle, TRZ: Trastuzumab, TRZ + NTB+DTX: Trastuzumab/Neratinib/docetaxel solution, TRZ−NP: Trastuzumab − Neratinib + Docetaxel conjugated nanoparticles.

The result of the cytotoxic effect of all formulations within the concentration range tested (1–10,000 nM), and at different time points, demonstrates a dose and time-dependent killing as shown in Figure 6 and in Supplement Figure S8.

**Figure 7:**
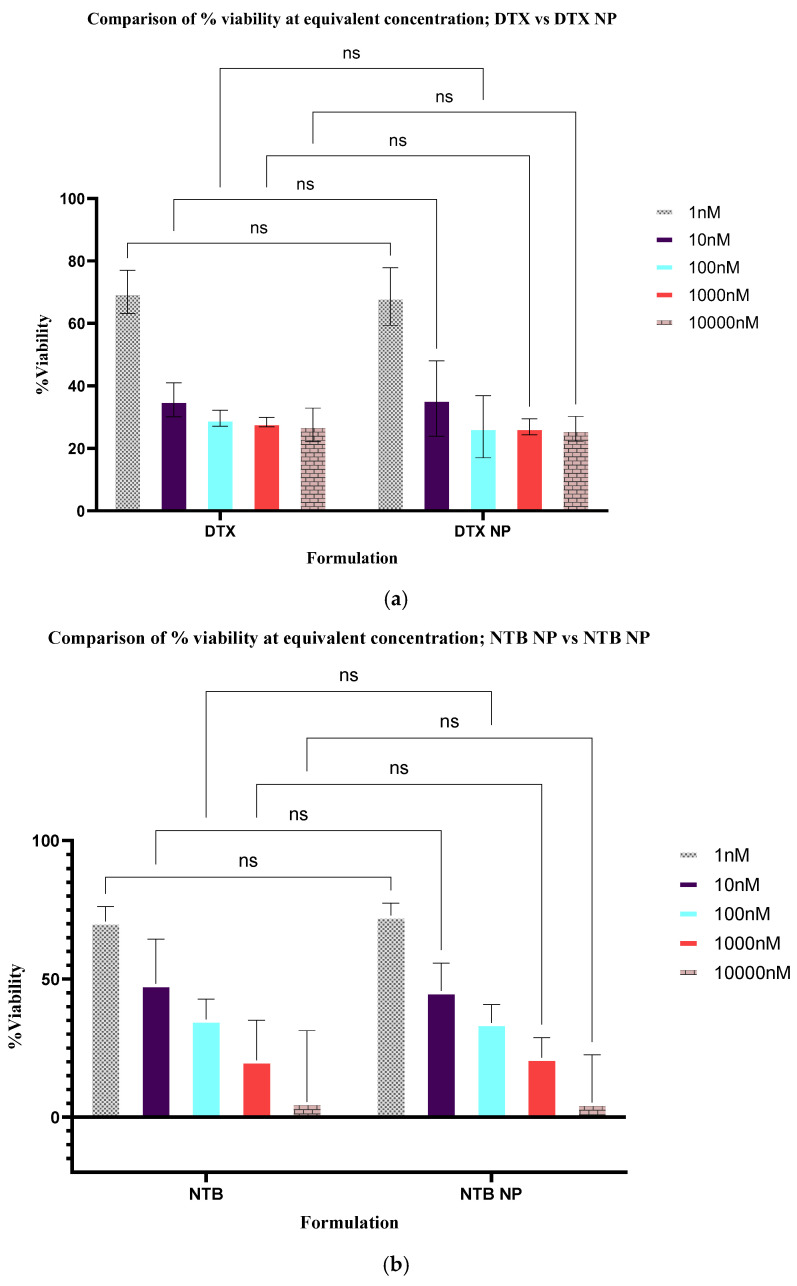

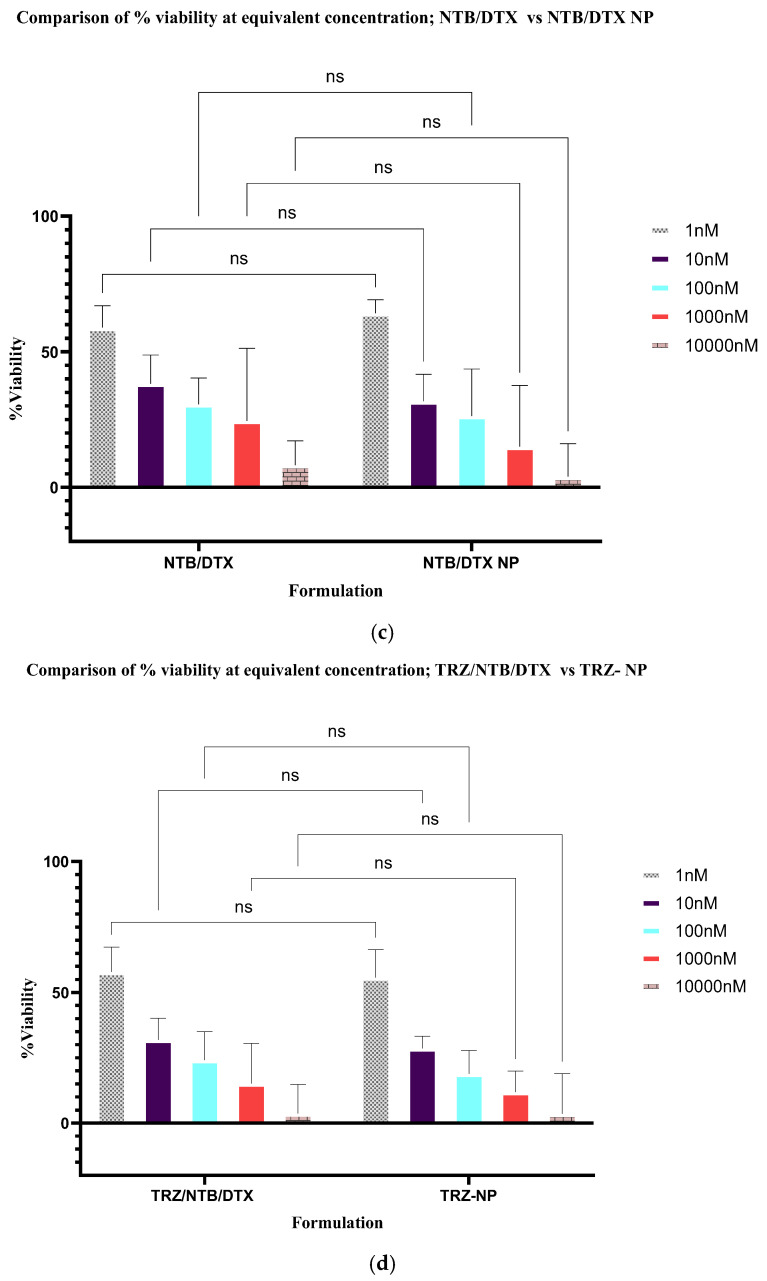

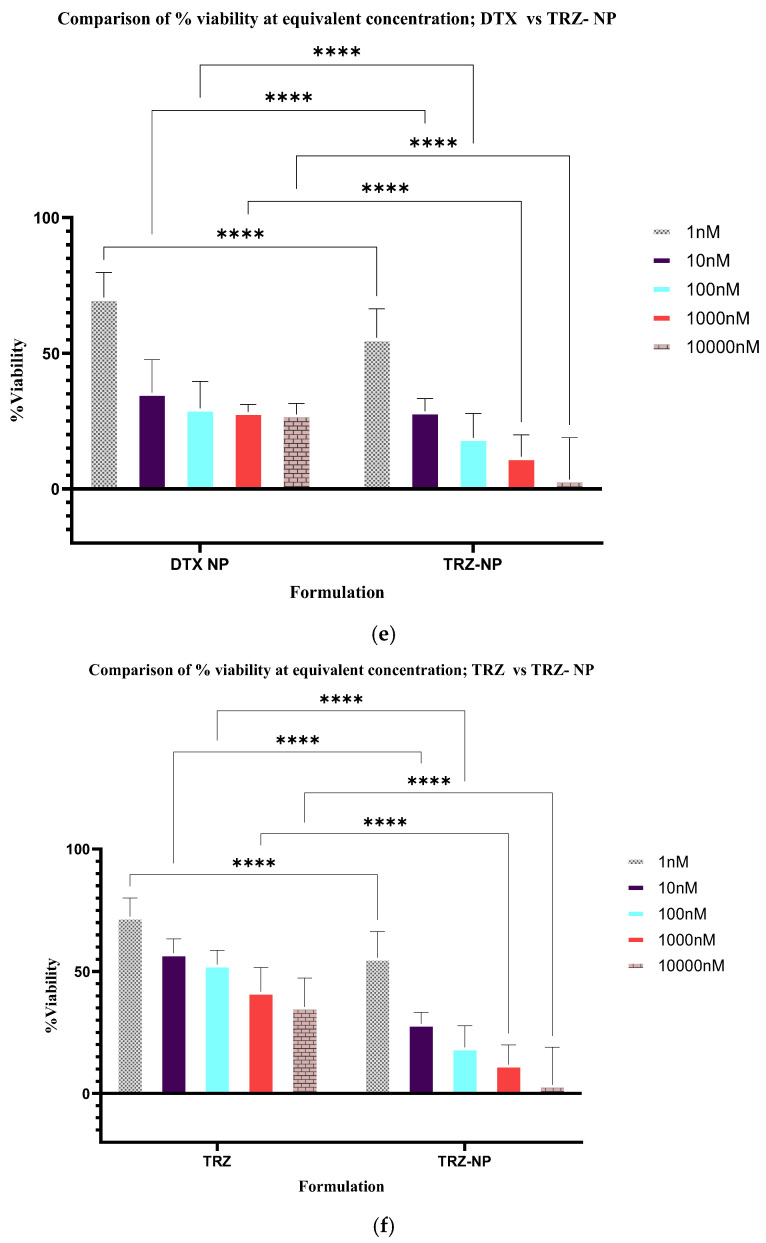
Comparison of % viability after treatment with (**a**) DTX vs. DTX−NP, (**b**) NTB vs. NTB−NP, dual solution of (**c**) NTB+DTX vs. DTX+NTB−NP, triple solution of (**d**) DTX+NTB+TRZ vs. TRZ−NP, (**e**) DTX−NP vs. TRZ−NP and (**f**) TRZ vs. TRZ−NP at 120 h. The asterisks correspond to statistical significance levels, with more asterisks indicating stronger significance (**** *p* < 0.00001, ns = not significant).

An analysis of variance (ANOVA) performed to evaluate the potential contribution of the culture medium, medium + DMSO, and blank nanoparticles to cytotoxicity (Supplement Figure S9) demonstrated no significant contribution from any of the three controls tested (*p* > 0.05). A two-way analysis of variance performed to evaluate any difference between the single drug solution and drug-loaded nanoparticles at different concentrations yielded no significant difference in viability for docetaxel (DTX) solution vs. DTX−NP (*p* = 0.999) and for neratinib (NTB) solution vs. NTB−NP (*p* = 0.9172) as shown in Figure 7a,b.

**Figure 8:**
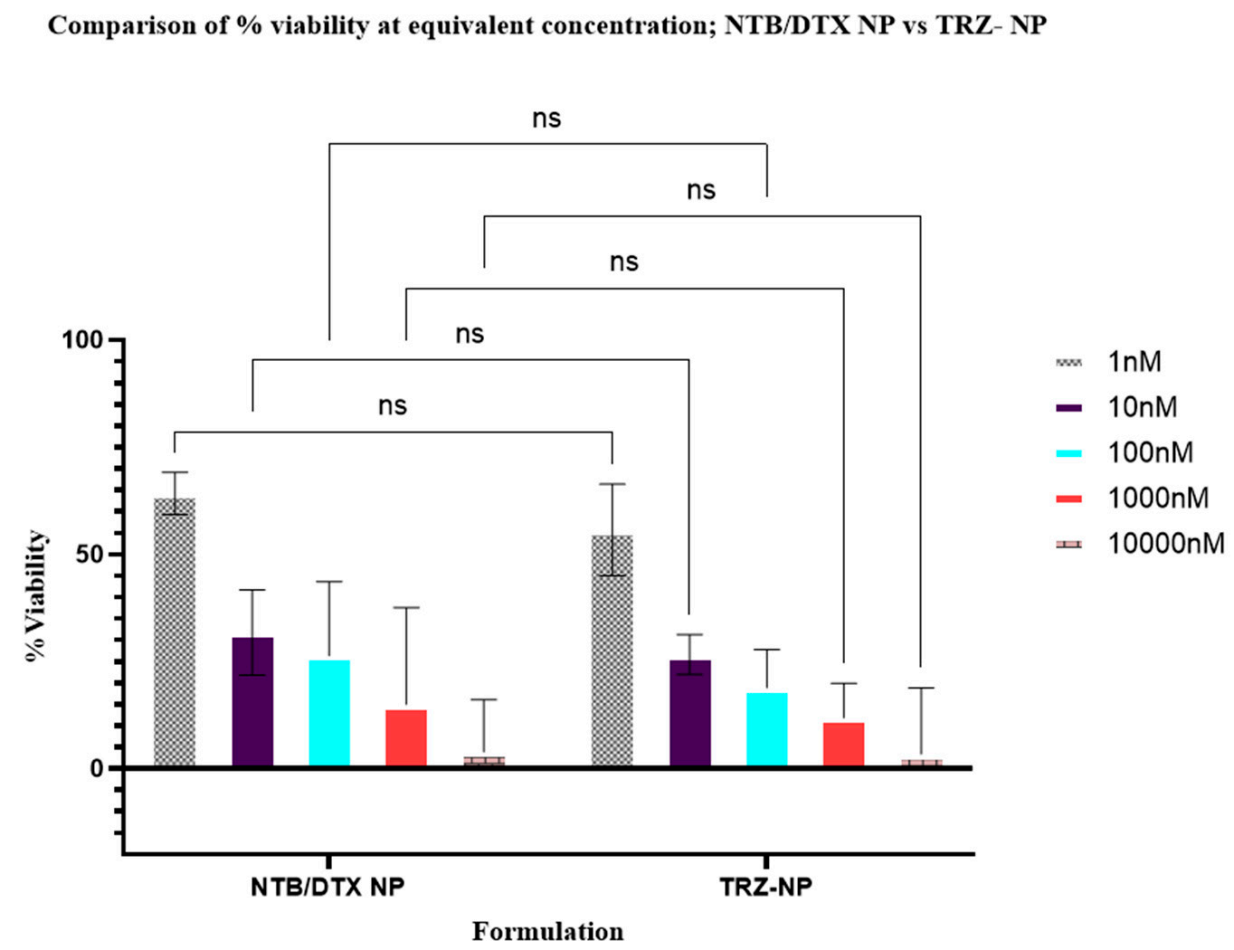
Comparison of % viability after treatment with DTX+NTB−NP vs. TRZ−NP, ns = not significant.

In another pairwise analysis, there was no statistical difference when the dual solution of neratinib and docetaxel was compared to DTX+NTB−NP (*p* = 0.9172); nor was there any difference between the solution of the combination of trastuzumab + neratinib + docetaxel and TRZ−NP (*p* = 0.6750). Analysis of the difference between TRZ−NP and other formulations showed a statistically significant reduction in the viability of SKBR-3 cells compared to pure trastuzumab (*p* < 0.001), NTB−NP (*p* = 0.0019), and DTX−NP (*p* = 0.0002) Figure 7). However, no significant difference in cell viability was observed between TRZ-NP and NTB+DTX−NP (*p* = 0.675), as shown in Figure 8. The enhanced efficacy of TRZ−NP compared to trastuzumab, NTB−NP, and DTX−NP is particularly noteworthy, given that TRZ−NP combines three drugs, each at half the concentration of its individual counterpart.

### 3.7. Evaluation of Combination Index (CI)

The combination index (CI) of neratinib and docetaxel, either in the dual (NTB+DTX− NP) or the conjugated (TRZ-NP) form, was analyzed using the Chou-Talalay method, a quantitative approach developed by Chou [46] and the result is presented in Table 1. IC_50_ values were derived via non-linear regression analysis (GraphPad Prism^®^, version 10.2.1) and from proprietary software (Compusyn^®^ version 2.0) to ensure consistency in results.

### 3.8. In Vivo Study

#### 3.8.1. Maximum Tolerated Dose

In the MTD (Maximum Tolerated Dose) studies, no mortality was observed at any of the administered doses throughout the study across all treatment groups. Additionally, there were no abnormal clinical observations during the experiments that required the censoring of animals. The dose escalation schedule is shown in Table 2.

**Figure 9:**
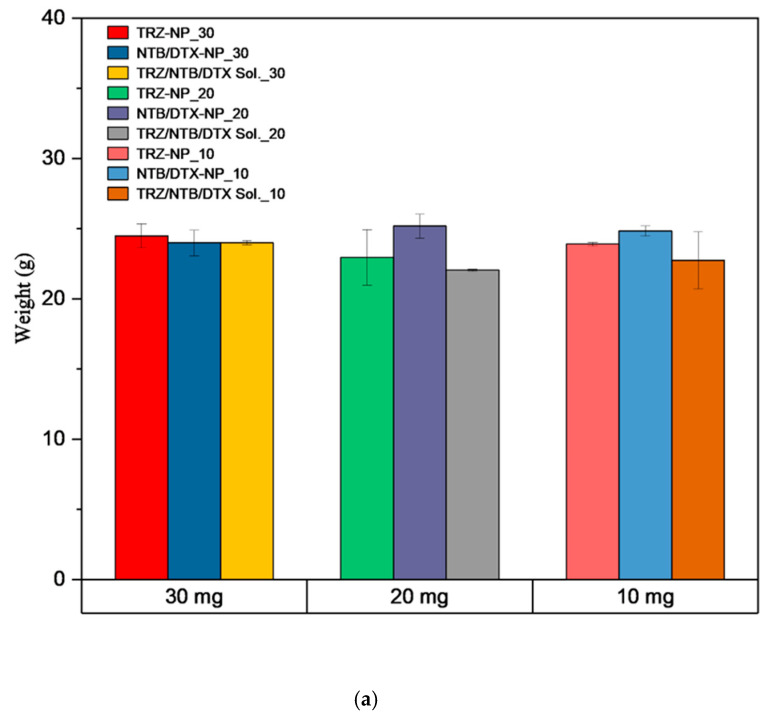

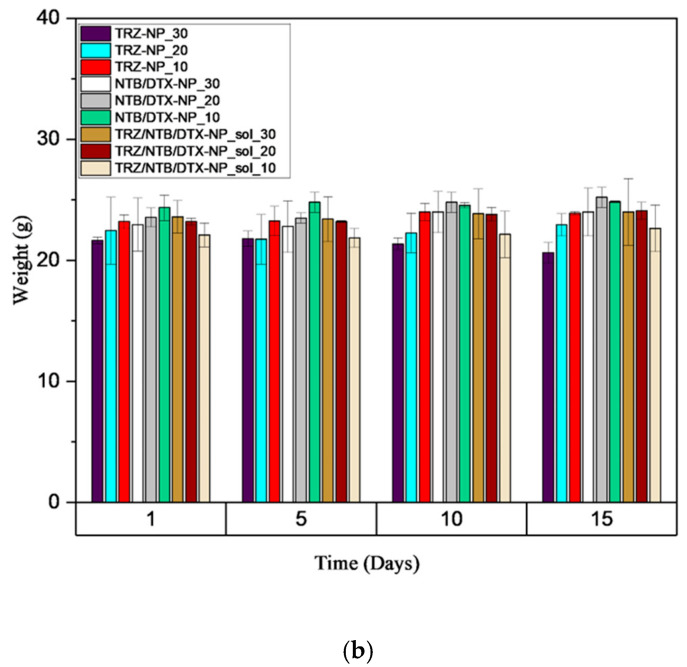
(**a**) Plot of the average changes in weight at all time points of mice exposed to escalating doses of different formulations. Weight was measured at four different time points. NTB+DTX−NP: Neratinib/docetaxel nanoparticle, and TRZ−NP: Trastuzumab−Neratinib + Docetaxel conjugated nanoparticles. (**b**) A two-dimensional bar plot showing the changes in weight of mice at the four time points. A 3D representation of the effect of treatment on weight with time is shown in Supplement Figure S11.

The mean body weight of mice receiving different doses of TRZ−NP, NTD+DTX−NP, and TRZ+NTB+DTX solutions was comparable within the period of observation, as shown in Figure 9a. Figure 9b shows another representation of the data, for clarity, by showing the changes in weight at different time points. Based on the tolerability observed in this toxicology study, the highest dose of 30 mg/kg was selected for subsequent efficacy studies. Parameters such as mortality, clinical signs, and body weight were carefully considered in determining the MTD.

#### 3.8.2. Efficacy Study

**Figure 10:**
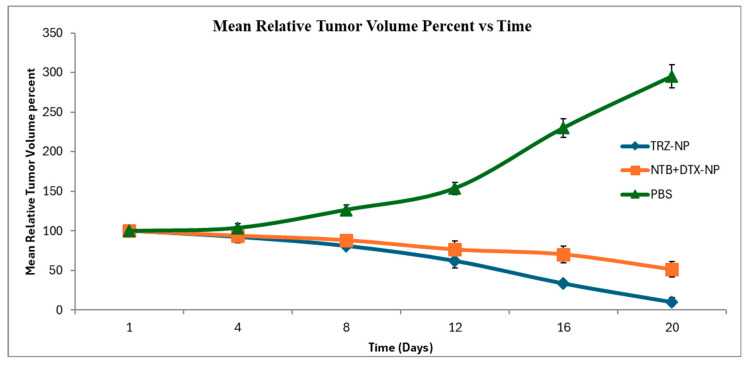
A line graph of mean relative tumor volume percent with time showing the change in tumor volume over the study period. Days 1, 4, 8, 12, 16 & 20 were the days data collection occurred.

Figure 10 shows the progressive decrease in mean relative tumor volume percent over time in the TRZ−NP group and the NTB+DTX−NP group. The PBS group with no drug intervention showed a strong progressive increase in mean relative tumor volume percent over time.

**Figure 11:**
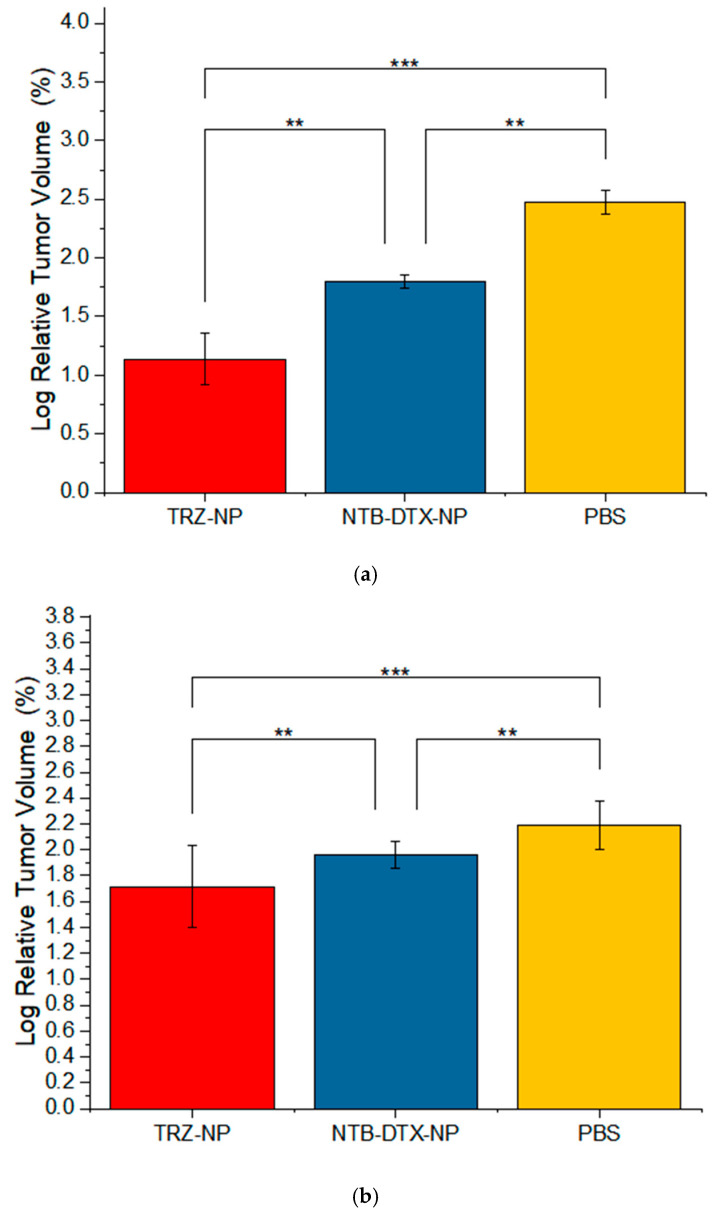
Pairwise comparison of Log Relative Tumor Volume (RTV %) for (**a**) Tumor volume measured on the last day of observation (Day 20) and (**b**) all observation points (Days 0–20). ** *p* ≤ 0.01, *** *p* ≤ 0.001.

Data collected on the last day of observation (relative tumor volume percent) were log-transformed and subjected to a one-way ANOVA to detect any differences among the three treatment groups. A multiple comparison post-hoc analysis (Figure 11a) demonstrates that TRZ−NP and NTB+DTX−NP significantly reduced tumor growth compared to the control group (PBS), respectively (*p* ≤ 0.001 and *p* ≤ 0.001). Pairwise analysis also demonstrated the superior tumor regressive effect of TRZ−NP compared to the unconjugated dual-loaded NTB+DTX−NP (*p* = 0.01).

To evaluate the robustness of our findings, we included all data points from the early phase of the study, where no apparent differences between groups were expected, and conducted a one-way ANOVA to assess any group differences. The results (Figure 11b) revealed a highly significant difference between TRZ−NP and NTB+DTX−NP (*p* = 0.01), NTB+DTX−NP and PBS (*p* = 0.01), and a strongly significant difference between TRZ−NP and PBS (*p* = 0.001).

**Figure 12:**
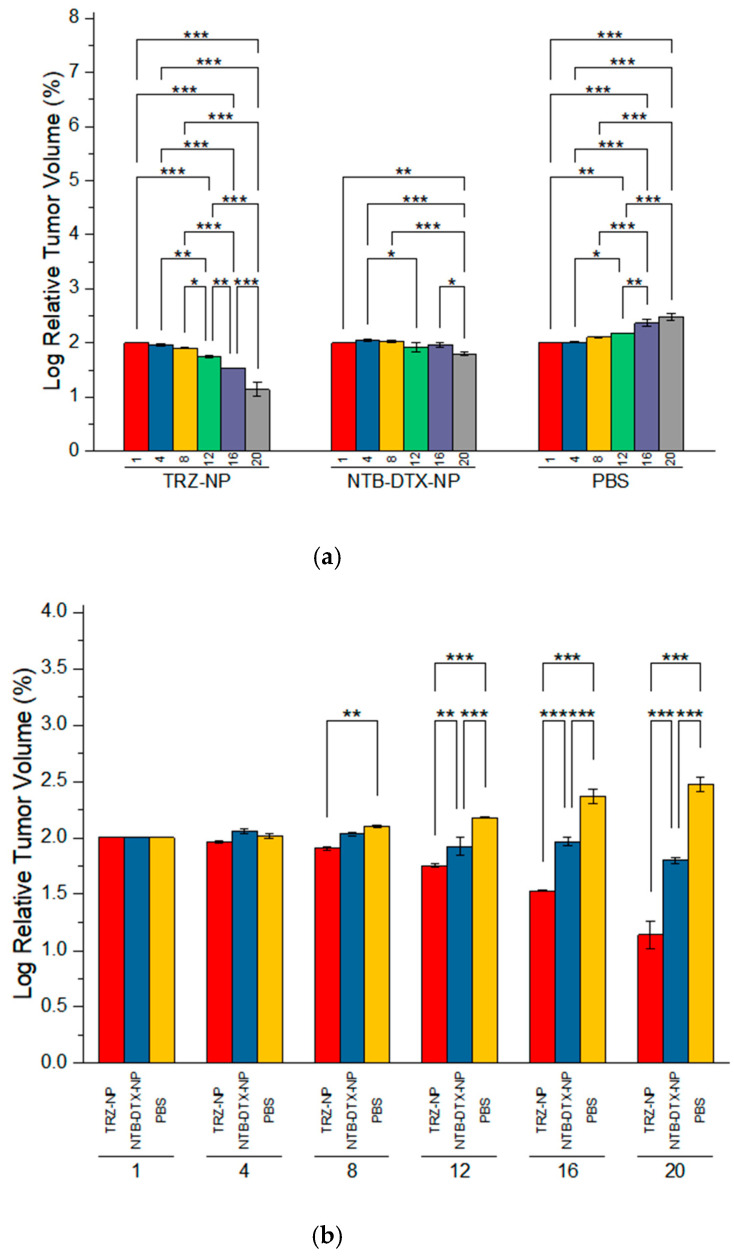
Pairwise comparison of log relative tumor volume with time (**a**) within groups and (**b**) between groups. * *p* ≤ 0.05, ** *p* ≤ 0.01, *** *p* ≤ 0.001.

In a two-way analysis of variance (Figure 12a), TRZ−NP demonstrated a highly significant reduction in tumor volume between most observation points compared to NTB+ DTX−NP. Conversely, the PBS group showed a significant increase in tumor volume over time. In another two-way analysis of variance between groups across different time points (Figure 12b), there was no difference between TRZ−NP, NTB+DTX−NP, and PBS on days 1 and 4 (*p* > 0.05). However, there was a progressive increase in the difference between groups at subsequent time points, culminating in a strongly significant interaction (PBS vs. TRZ−NP, PBS vs. NTB+DTX−NP, and TRZ-NP vs. NTB+DTX−NP; *p* ≤ 0.001) at the end of the study.

Although NTB+DTX−NP reduced tumor growth substantially and comparably (*p* ≤ 0.01) to TRZ−NP in the pairwise comparison with PBS towards the end of the study (Days 16 & 20), however, TRZ−NP was superior overall in regressing tumor volume either early in the treatment cycle or at the end of the study (TRZ−NP vs. NTB+DTX−NP, *p* ≤ 0.001).

In this study, tumor growth inhibition (TGI) (%) was computed with the formula

(3) $$\frac{\mathrm{TGI}\;\left(\%\right)=[1\;-\;\left(\mathrm{RTV}\;\mathrm{of}\;\mathrm{the}\;\mathrm{treated}\;\mathrm{group}\right)}{\left(\mathrm{RTV}\;\mathrm{of}\;\mathrm{the}\;\mathrm{control}\;\mathrm{group}\right)]\;\times 100\;\left(\%\right)}$$

where RTV = Relative tumor volume, TGI = Tumor growth inhibition.

**Figure 13:**
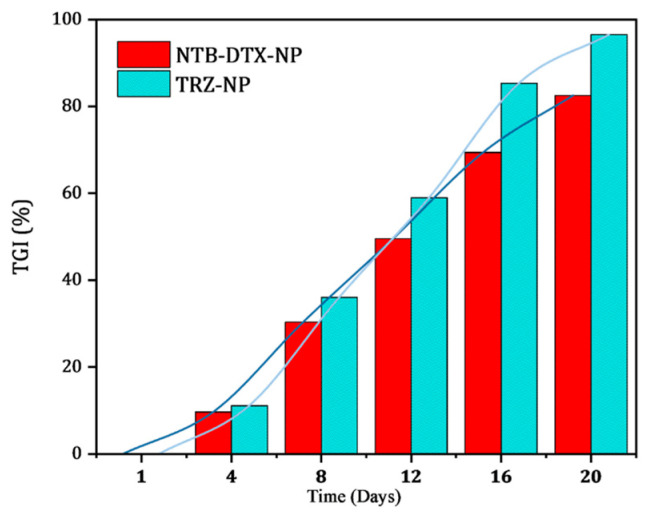
Tumor growth inhibition ratio with time.

The data generated was plotted against time (days) as shown in Figure 13. TRZ-NP demonstrates a better tumor growth inhibitory effect compared to NTB+DTX−NP over time.

## 4. Discussion

The identification of the HER2 (human epidermal growth factor receptor) receptor, its critical role in breast cancer tumorigenesis, and its targeting with monoclonal antibodies have significantly revolutionized the treatment of HER2-positive cancers [37]. The standard therapy involves a dual monoclonal antibody regimen combining trastuzumab and pertuzumab with cytotoxic agents, predominantly taxanes, designed for synergistic efficacy and minimal resistance [47]. Despite these advancements, several challenges persist in the treatment of HER2-positive breast cancer. These include the toxicity of non-targeted cytotoxic drugs [48], the cardiotoxic side effects of pertuzumab that limit its long-term use, and the grade 3/4 gastrointestinal side effects associated with pertuzumab and neratinib [49]. Additionally, the mode of administration of these drugs can be inconvenient, and their non-synchronized pharmacokinetic profiles often result in uneven arrival at the biophase (site of action), potentially contributing to drug resistance. Furthermore, the dosing of small-molecule tyrosine kinase inhibitors typically involves administering several oral tablets daily, which can pose additional challenges in ensuring patient compliance in the effective management of HER2-positive breast cancer.

This study developed a multifunctional, trastuzumab-conjugated nanoparticle system for HER2-targeted co-delivery of neratinib and docetaxel, designed to achieve: (1) enhanced tumor-specific accumulation, (2) controlled dual-drug release kinetics, (3) mitigation of resistance mechanisms (e.g., P-glycoprotein efflux), and (4) synergistic anticancer activity.

Nanoparticles were formulated from PLGA surface-decorated with PEG and derivatized with COOH groups. Dual (neratinib & docetaxel), single (neratinib or docetaxel), and blank nanoparticles were formulated by nanoprecipitation according to a previously published report [22]. Nanoprecipitation is a simple, energy-efficient method for fabricating nanoparticles (NPs) with tailored properties and functionalities [50]. Unlike other techniques requiring high shear forces (ultrasonication) or the use of surfactants for emulsification, nanoprecipitation achieves robust NP formation without these additional complexities. Moreover, structural features critical for other emulsification techniques, like amphiphilic character or ionic charges, are less significant in this process [51]. This versatility makes nanoprecipitation compatible with a broad range of polymers, including commonly used hydrophobic synthetic homopolymers such as polystyrene (PS) and poly (methyl methacrylate) (PMMA), as well as biodegradable polymers like poly(ε-caprolactone) (PCL), poly(lactide) (PLA), and poly(lactide-co-glycolide) (PLGA) [52,53]. Therefore, the use of PLGA-PEG for nanoparticle formulation in this study is both practical and well-suited to the desired outcomes. The long-term stability of nanoparticles is a crucial consideration in the choice of methods for nanoparticle fabrication. It has been reported that a key advantage of nanoprecipitation is the ability to tune the dimensions of NPs from several tens of nanometers to sub-micron size [54]. Consequently, homogeneous dispersions prepared by nanoprecipitation usually exhibit high stability for days or even months without the need for surfactants [55]. Further, PLGA and PEG have been approved for use in humans by the FDA.

The conjugation of trastuzumab to drug-loaded nanoparticles followed a previous method used in our laboratory [23]. The first step in this process is the oxidation of carbohydrate residues (mannose, galactose and N-acetyl glucosamine) on the FC region of trastuzumab to convert them to aldehydes. The presence of aldehyde groups was verified with Purpald’s assay (purple color). This method is non-destructive and offers superior sensitivity compared to other techniques, making it ideal for the assay of thermolabile biologics [41]. The presence of aldehyde groups was further verified by UV analysis of a sample of oxidized trastuzumab at the formaldehyde absorption wavelength of 550 nm.

Hydrazines such as carbohydrazide are used as carbonyl derivatizing agents because of the ease with which they form conjugates with aldehydes and ketones [27]. They form hydrazone bonds with carbonyl groups that are typically detected via UV or fluorescence analysis. In this experiment, the hydrazone bond formed between the aldehyde groups on trastuzumab and carbohydrazide was reduced to a stable hydrazine bond with NaCNBH_3_ [26]. The initial hydrazone bond was verified with UV analysis at a wavelength of 290–400 nm [27,28]. A comparison of the UV spectrum of TRZ−carbohydrazide, oxidized trastuzumab, and PBS under similar conditions is shown in Supplement Figure S2. The stacked spectrometric analysis clearly shows the superior absorption of the hydrazone-containing sample (TRZ−carbohydrazide) compared to other samples tested within this range. The formation of a hydrazine bond between carbohydrazide and the aldehyde group on trastuzumab was further characterized with FTIR, as shown in Supplement Figure S3. The lower spectrum of TRZ-carbohydrazide shows the typical N-N stretch of hydrazine conjugation at 1071 cm^−1^, indicating a bond formation with trastuzumab. This N-N stretch was not observed in the pure trastuzumab sample, as shown in the top spectrum.

Coupling of trastuzumab to nanoparticles was evaluated by centrifugation based on the assumption that only molecules of trastuzumab-carbohydrazide coupled to nanoparticles would sediment under force, while the uncoupled trastuzumab-carbohydrazide would remain suspended [30,42,43]. FTIR analysis of TRZ−Carbohydrazide before conjugation to nanoparticles and the supernatant (uncoupled trastuzumab-carbohydrazide) after conjugation to nanoparticles both display similar peak signatures. For instance, both samples display a broad peak in the 3200–3600 cm^−1^ region, which corresponds to the stretching vibrations of hydroxyl (O-H) and amide (N-H) groups (Supplement Figure S4). These peaks suggest the presence of hydroxyl and amide functional groups in both TRZ-carbohydrazide and the supernatant. Secondly, the band near 1640 cm^−1^, typical for amide groups, appears in both spectra, indicating a consistent chemical structure between the two samples. Finally, a significant peak in the region near 1015–1077 cm^−1^, significant for N-N stretching, is present in both spectra, indicating similarity between both samples [56] and suggesting that most of the nanoparticles formed pellets after centrifuging and are not present in the supernatant.

Further, comparison of the starting blank nanoparticles with TRZ−NP highlights a series of modifications, as illustrated in Supplement Figure S10. The blank nanoparticles (top spectrum) display a prominent peak at 1754.99 cm^−1^, characteristic of carbonyl (C=O) stretching vibrations typically associated with esters or amides. Peaks between 1451–1170 cm^−1^ correspond to C-H bending and C-O stretching, features common in most organic compounds. Additionally, a smaller peak at 1089 cm^−1^**,** attributable to C-O-C vibrations, indicates the presence of ether groups. In contrast, TRZ−NP (bottom spectrum) demonstrates notable changes. A broad peak around 3364 cm^−1^ suggests O-H stretching, likely due to hydroxyl groups or hydrogen bonding. The C=O stretching peak at 1754.99 cm^−1^ remains, but new peaks emerge at 1639 cm^−1^**,** indicative of amide linkage formation or hydrogen-bonded carbonyl groups. The region between 1250–1000 cm^−1^ features additional peaks that point to ether or other C-O stretching vibrations. The integration of the broad O-H stretches at 3364 cm^−1^, observed in pure trastuzumab, with the characteristic peaks of blank nanoparticles in TRZ−NP, supports the assertion of successful conjugation between the two entities. This conjugation combines the functional groups of trastuzumab with those present on the nanoparticle surface.

The conjugation of TRZ to nanoparticles was further validated with BCA assay based on the absorbance intensities of various samples: BCA, dual drug-loaded nanoparticles, TRZ−NP, and pure TRZ, between 560–600 nm wavelengths. Since TRZ−NP went through multiple wash steps, any absorbance thereafter was attributed to the presence of TRZ in the nanoparticle conjugate. As shown in the UV spectra (Figure 4), differences in peak heights among the samples reflect variations in protein concentration. Notably, the peak absorbance of TRZ−NP and pure TRZ at 562 nm is closely matched, suggesting their concentrations are almost equivalent, thus validating the conjugation of TRZ to nanoparticles. The minimal interference observed in the lower wavelength region (200–400 nm) underscores the specificity and reliability of the BCA assay.

In this study, the ~5% trastuzumab conjugation observed represents a low-to-moderate surface coverage that remains within the functional range reported in the literature [57,58,59]. Previous studies indicate that optimal targeting is achieved at an intermediate ligand density, as excessively high antibody loading can reduce binding efficiency, alter biodistribution, and trigger unwanted immune responses [60,61,62,63]. Effective receptor-specific binding and internalization have been demonstrated even at modest antibody densities when orientation and avidity are favorable [64]. Our in vitro and in vivo results support the functional adequacy of the current conjugation level, and future work will explore tuning antibody density to optimize targeting performance.

Following fabrication, the nanoparticles were characterized. To leverage the Enhanced Permeability and Retention (EPR) effect and maximize tumor accumulation, the optimal nanoparticle size has been suggested to range between 10 and 300 nm [65]. In this study, blank nanoparticles had an average size range of 87.4 nm to 95.8 nm; while the dual-loaded nanoparticles ranged from 156.3 nm to 166.4 nm. Single docetaxel and neratinib-loaded nanoparticles demonstrated sizes that ranged from 129.7 nm to 145 nm. The particle size of the dual-loaded nanoparticles increased to a range of 261.3 nm to 296.6 nm after conjugation with the targeting moiety, trastuzumab. Despite these increases, the particle sizes remained within the optimal range to leverage the EPR effect, which is recognized as the hallmark of both passive and active tumor-targeting strategies [66,67].

A one-way ANOVA (analysis of variance) with multiple comparison test was performed to evaluate the impact of single and dual loading, as well as the effect of trastuzumab conjugation on the particle size of the various nanoparticles. The evidence shows that as drug molecules are incorporated into the nanoparticle matrix, they contribute to the overall mass and volume of the nanoparticles [68]. These results demonstrate an increase in the size of the nanoparticles with drug and targeting moiety conjugation and highlight statistical differences (*p* < 0.05) between groups.

A high zeta potential (positive or negative) indicates good stability, as repulsive forces between particles prevent aggregation [69]. In applications like nanoparticle drug delivery, zeta potential influences particle behavior in biological systems, including interaction with cells and proteins [70].

In this study, the zeta potential of the various nano-formulations ranged from approximately −15 mV to −20 mV, indicating negatively charged surface characteristics across board. To evaluate the impact of single and dual loading as well as the effect of trastuzumab conjugation on the zeta potential of the various nanoparticles, a one-factor ANOVA with multiple comparison test was performed. An assessment of the effect of drug loading shows that neither single, dual-loading (DTX, NTB, or both) nor conjugation with trastuzumab (TRZ−NP) appears to significantly affect the zeta potential compared to the blank NP formulation. This observation suggests that the sequential modification of this delivery system does not drastically alter its surface charge properties. The uniform zeta potential across all formulations indicates consistent surface charge characteristics, with no significant impact from drug loading or targeting moiety conjugation. This stability is advantageous for ensuring reproducibility and uniformity in biological interactions and delivery. While higher absolute values (e.g., beyond ±30 mV) typically indicate stronger electrostatic repulsion and greater stability, the values reported here are still outside the zwitterionic/neutral zone of ±10 mV, which are prone to agglomeration [71]. Hence, these nanoparticles are suitable for subsequent experiments.

The in vitro release isotherm of both NTB and DTX in the dual-loaded formulation shows an initial increase within the first 50 h, followed by a gradual plateau after approximately 150 h and 200 h, respectively, approaching 100% cumulative release, and suggesting that the bulk of both drugs is released over this time frame. Drug loading was found to be 18.81% for neratinib and 6.07% for docetaxel. In the conjugated nanoparticle, maximum drug release was observed at 120 h, while % drug loading was calculated to be 8.49% for neratinib and 1.25% for docetaxel. The conjugation process led to a 45% and 20.5% decrease in drug loading for neratinib and docetaxel, respectively, compared to the dual-loaded nanoparticles. The release profile showed an average release period of 8 days in the dual-loaded formulation. Therefore, to confirm the stability of analytes released from the nanoparticles, LC-MS analysis (Supplement Figure S12) was performed on a sample collected on the 8th day of drug release, and the result confirmed the presence of both neratinib and docetaxel, an indication that these analytes are stable within the drug release matrix over this period.

While this study demonstrated short-term stability of the released analytes over an 8-day period, long-term stability of the intact nanoparticle–antibody conjugates was not assessed. This work was designed as a proof-of-concept evaluation, with the primary aim of establishing formulation feasibility and therapeutic potential. Nonetheless, extended stability profiling-including monitoring particle size, zeta potential, antibody binding activity, and drug retention over prolonged storage under various conditions—will be essential for advancing this platform toward translational application. In addition, scalability and reproducibility of the synthesis will require systematic process optimization and batch-to-batch consistency assessments under controlled manufacturing conditions. These aspects are planned for future studies to support preclinical development and potential regulatory submission.

A binding affinity study was performed to evaluate the preliminary biological activity of TRZ−NP conjugates [23]. Statistical analysis suggests there is no dramatic loss of binding affinity to HER2-positive cells due to the conjugation of nanoparticles to trastuzumab (*p* = 0.505). Conversely, there was a significant difference in the binding affinity of TRZ vs. HIgG1 (*p* = 0.01) and TRZ−NP vs. HIgG1 (*p* = 0.04), largely due to the poor affinity of HIgG1 for the biomarker (HER2 receptors). Since HIgG1 lacks the capacity to bind to HER2-positive receptors, it is unable to bind the antihuman FITC-conjugated antibody effectively. Hence, the low FITC fluorescence event in the control group [72,73] as shown in Figure 6.

Cellular uptake of the nanoparticles was analyzed using confocal laser scanning microscopy (CLSM) [33]. The results demonstrate that discrete nanoparticle uptake was evident within 6 h of exposure. The images in Supplement Figure S7 reveal distinct staining patterns for different cellular components. The nucleus, stained blue with Hoechst dye, is visible in the top right quadrant [74]. The plasma membrane is highlighted in red with CellMask^®^ staining in the bottom left quadrant, while rhodamine-123, emitting green fluorescence, is shown in the top left quadrant. These nanoparticles are localized around the nucleus and confined within the boundary of the plasma membrane. The merged images, presented in the bottom right quadrant, illustrate the spatial relationships between the nucleus, cytoplasm, and plasma membrane. The images provide clear evidence of nanoparticle localization within the cell membrane boundary at different time points. Some nanoparticles are seen adhering to the plasma membrane, while others are in the process of being internalized into the cytoplasm. This progression indicates effective cellular uptake, with increasing nanoparticle internalization over time (6, 24, and 48 h).

The results of the antiproliferative studies suggest that, within the concentration range tested (1–10,000 nM), all formulations demonstrated dose and time-dependent cytotoxicity on SKBR3 cells (Supplement Figure S8a,b) compared to controls (medium, medium + DMSO & blank nanoparticles).TRZ−NP exhibited superior cytotoxicity vs. single-drug NPs (NTB−NP: *p* = 0.0019; DTX−NP: *p* = 0.0002) and free TRZ (*p* < 0.001), despite containing half the individual drug concentrations. The superior effect of the dual and conjugated nanoparticles at half the concentration of the individual agents across various formulations and time indicates a synergistic or additive relationship. The Chou-Talalay combination index (CI) analysis (Table 1) supports the theory of synergism (CI < 1) between NTB, DTX, and TRZ in both dual-loaded and conjugated NPs. The lack of observed enhancement in biological activity with the addition of the monoclonal antibody [TRZ−NP and DTX+NTB−NP (*p* = 0.675)] in TRZ−NP may be attributed to the absence of effector cells required for its cell-mediated cytotoxic effect under in vitro conditions, rather than a lack of pharmacological synergy or additive efficacy. A similar outcome was reported by Abedin et al., where mean cell death induced by trastuzumab-conjugated paclitaxel nanorods did not differ significantly from that of single-agent treatments [75]. This data set reinforces the advantages of nanoparticle-based delivery systems in enhancing cytotoxicity, likely due to improved drug stability, controlled release, and targeted delivery.

Our trastuzumab-conjugated dual-loaded neratinib/docetaxel nanoparticles exhibited the lowest IC_50_ (1.432 nM) compared with the dual solution (3.013 nM) and non-targeted nanoparticles (3.102 nM), representing ~54% enhanced potency over non-targeted formulations. This trend aligns with prior reports of cytotoxicity in SKBR-3 cells. Mandala et al. reported similar IC_50_ values for non-targeted neratinib/docetaxel systems (3.327–3.547 nM) but did not assess trastuzumab targeting [76]. Abedin et al. showed targeting benefits in paclitaxel nanorods (IC_50_: 31 nM vs. 54 nM in solution), though their potencies were ~20-fold lower than ours [75]. Aleanizy et al. demonstrated that trastuzumab-grafted neratinib-loaded dendrimers achieved greater cytotoxicity against SKBR-3 cells (33% viability) than both neratinib-loaded dendrimers (36% viability) and free neratinib (40% viability) at 45 ng/mL, highlighting the incremental benefit of HER2 targeting [77]. Chih-Sheng Chiang et al. reported that trastuzumab-conjugated dual loaded paclitaxel-doxorubicin double emulsion nanocapsules (PTX-DOX-DENCS) achieved the greatest cell killing (12.4% viability), consistent with our finding that active targeting combined with dual-drug delivery yields maximal potency [78]. Overall, our system ranks among the most potent HER2-targeted dual-drug nanoformulations reported to date.

The MTD study demonstrated an excellent safety profile across all treatment arms, with no mortality or life-threatening adverse events observed at any of the administered dose levels. Clinical observations revealed that animals receiving free drug combinations (docetaxel, neratinib, and trastuzumab) exhibited more pronounced toxicity-related symptoms, including skin exfoliation, macular changes, generalized weakness, and behavioral isolation, compared to animals treated with their respective nanoformulations. These findings are consistent with the known systemic toxicities of taxanes and irreversible tyrosine kinase inhibitors when administered as free molecules. The nanoformulation significantly mitigated these toxicities, likely due to improved pharmacokinetic behavior, altered biodistribution, and a controlled drug-release profile.

Based on a comprehensive assessment of clinical signs, mortality, and body weight trajectories, a dose of 30 mg/kg was established as the MTD for subsequent efficacy evaluation. Importantly, all animals were humanely euthanized per IACUC protocol, further confirming that the defined MTD was non-lethal and ethically appropriate for downstream efficacy studies.

The efficacy arm of this investigation utilized a HER2-positive SKBR-3 breast cancer xenograft model in athymic nude mice to evaluate the therapeutic impact of dual-loaded nanoformulation (NTB+DTX−NP) and targeted trastuzumab-conjugated nanoparticles (TRZ−NP) compared to vehicle control (PBS). Power was calculated using G*Power 3.1.9.7 with a medium effect size, α = 0.05, and a repeated-measures ANOVA (tumor size assessed at six time points) approach. For a total sample size of 18 distributed across three groups, the calculated power was 0.79. Both TRZ−NP and NTB+DTX−NP demonstrated significant tumor growth inhibition relative to the control, affirming the effectiveness of nanoparticle-based delivery in enhancing therapeutic outcomes. Notably, TRZ−NP exerted a superior and sustained reduction in tumor burden over time, compared to NTB + DTX−NP. This trend was evident both at early treatment points and at the terminal observation time point, as measured by relative tumor volume (% RTV) and supported by robust statistical significance (TRZ−NP vs. NTB+DTX−NP, *p* ≤ 0.001). Given the equivalent dosing across both nanoparticle formulations, the enhanced antitumor effect of TRZ−NP likely stems from its targeted delivery mechanism and engagement of antibody-dependent cellular cytotoxicity (ADCC), which is particularly relevant in HER2+ tumors. These observations are consistent with the mechanism of action of trastuzumab, which not only inhibits HER2-mediated signaling but also recruits immune effector cells through its Fc domain to mediate ADCC. However, the absence of immune cells in certain in vitro conditions could obscure this benefit, underscoring the importance of an intact immune microenvironment in realizing the full therapeutic potential of mAb-based strategies. The use of athymic nude mice, although lacking T-cells, retains sufficient innate effector cell populations (e.g., NK cells) to facilitate partial ADCC, allowing some degree of immune-mediated tumor clearance.

One-way ANOVA with post-hoc comparisons confirmed the significant tumor inhibitory effects of both TRZ-NP and NTB+DTX−NP relative to PBS (each *p* ≤ 0.001). Furthermore, direct pairwise analysis between the two active treatment groups demonstrated the superior efficacy of TRZ−NP (*p* = 0.01), reinforcing the conclusion that targeted delivery, rather than dose alone, drives the differential efficacy. Time-course analysis using two-way ANOVA further validated the robustness of these effects, showing that while no early differences were detectable on Days 1 and 4, progressively significant divergences emerged by later time points (Days 16 and 20), suggesting cumulative therapeutic impact over multiple dosing cycles. This time-dependent therapeutic gain is further corroborated by the tumor growth inhibition (TGI) analysis, wherein TRZ−NP exhibited consistently superior inhibition compared to NTB+DTX−NP. The difference in TGI trajectories highlights not just improved drug accumulation at the tumor site, but also a potential amplification of therapeutic effect via antibody-mediated immune recruitment.

Previous in vivo reports have shown that HER2-directed nanocarriers improved therapeutic outcomes over non-targeted or standard controls. Chiang et al. demonstrated that trastuzumab-conjugated PTX−DOX−DENCS carriers, when combined with magnetic targeting, achieved the lowest relative tumor volume compared to the unconjugated formulations [78]. Peng et al. likewise showed that Herceptin conjugated paclitaxel loaded polycaprolactone-PEG nanoparticles (PTX@PCL-PEG-Herceptin) suppressed tumor growth by >2–3 folds versus TAXOL^®^ and non-targeted PTX@PCL-PEG [79]. PTX@PCL-PEG-Herceptin was also found to be superior by 3.0, 2.4, and 3.4 folds versus Saline, Herceptin, and Blank micelles over 48 days. Chia-Yu Su reported a tumor inhibitory rate of 88.9% for HER2-LsbMDDs (bispecific antibody conjugated drug-loaded lecithin-stabilized micellar drug delivery system), exceeding all comparators (85.8–80.1%) [80]. Despite differences in drug payloads and readouts (tumor volume, relative tumor volume, inhibitory rate), which call for cautious interpretation of cross-study comparisons, the directional concordance across studies supports our conclusion that active HER2 targeting, particularly when combined with dual-drug delivery, enhances in vivo antitumor efficacy beyond non-targeted nanoformulations.

Several studies in the literature have explored the design of dual-loaded nanoparticle conjugates; however, none mirrors the specific approach presented in this work. For example, Heggannavar et al. developed a dual-loaded nanoparticle system by combining docetaxel-loaded mesoporous silica nanoparticles with paclitaxel-loaded PLGA nanoparticles using a double emulsion (solid-in-oil-in-water) solvent evaporation technique, followed by conjugation with Angiopep-2 to target low-density lipoprotein receptor-related protein 1 (LRP-1) [81]. While there are parallels with our study in the use of PLGA and docetaxel, key distinctions include the nanoparticle formulation method, incorporation of polyvinyl alcohol (PVA)—a surfactant associated with cytotoxicity, and the selection of a different targeting ligand. In another study, Carvalho et al. encapsulated ABT-737 and Purvalanol into PEGylated PLGA nanoparticles, which were subsequently conjugated to gemtuzumab for targeting the CD33 transmembrane protein in pediatric acute myeloid leukemia (AML) [82]. Although the use of PEG-PLGA aligns with our formulation, differences are evident in the combination of therapeutic agents, nanoparticle fabrication methodology, surfactant use (PVA), and targeting ligand. Finally, a recent report from our group described the formulation of dual-loaded nanoparticles containing paclitaxel and cisplatin using PEGylated methacrylate–polylactide copolymer, conjugated with cetuximab for triple-negative breast cancer (TNBC) therapy. While this platform shares a similar formulation approach with the current study, it differs in the choice of polymer, therapeutic payloads, and targeting agent [83].

## 5. Conclusions and Implications for Further Development

Taken together, these findings provide compelling preclinical evidence that TRZ−NP offers a mechanistically enhanced and better-tolerated approach to HER2+ breast cancer therapy compared to either the free drugs or the non-targeted dual-drug nanoparticle systems. The tolerability data justify the selected MTD of 30 mg/kg, and the efficacy results underscore the clinical promise of integrating targeted delivery with immunologic engagement. Further investigation in immunocompetent models or humanized mouse systems may more fully capture the ADCC potential of TRZ−NP. Additionally, pharmacokinetic and biodistribution studies will be instrumental in correlating tumor uptake with therapeutic outcome and eventually informing rational dose translation for clinical trials. To the best of our knowledge, this study is the first to develop a nanoplatform that encapsulates docetaxel and neratinib in nanoparticles conjugated with trastuzumab for targeted delivery to HER2+ tumor cells.

## Figures and Tables

**Table 1:** Summary of IC_50_ and combination index of formulations (Unit of IC_50_ values are in nM).

	120 h				72 h			
Formulation	IC50 calc.	Compu syn IC50	CI	Interaction	IC50 calc.	Compu syn IC50	CI	Interaction
Docetaxel sol.	5.805 ± 2.91	5.80012			250.8 ± 246	334.356		
Docetaxel NP	4.55 ± 4.08	4.04336			183.5 ± 102.74	274.694		
Neratinib sol.	11.91 ± 2.97	12.3085			331.9 ± 153.09	210.166		
Neratinib NP	11.69 ± 4.55	12.506			347.2 ± 179.23	253.618		
Neratinib/Docetaxel sol.	3.013 ± 1.88	3.58186	0.58614	+++	66.17 ± 37.97	72.7428	0.28184	++++
Neratinib/Docetaxel NP	3.102 ± 0.46	3.64065	0.59576	+++	108.6 ± 76.92	123.13	0.46687	+++
Trastuzumab sol.	166.8 ± 47.53	174.091			2315 ± 772.52	2273.08		
Trastuzumab Neratinib/Docetaxel sol.	1.938 ± 0.46	2.41153	0.20852	++++	14.78 ± 7.41	14.9498	0.04081	++++
Trastuzumab conjugated dual loaded NP	1.432 ± 1.88	1.50053	0.16657	++++	13.96 ± 3.47	14.7652	0.03949	++++
IC50 = Inhibitory concentration	Combination index Key:			<1 = Synergistic effect	++++ = Very strong synergism			
NP = nanoparticles				>1 = Antagonistic effect	++++ = Strong synergism			
CI = Combination index				1 = Additive effect	+++ = Synergism			

**Table 2:** Dose schedule for MTD study.

S/N	Groups	Dose
1	Trastuzumab-dual loaded nanoparticles	30 mg/kg
2	Trastuzumab-dual loaded nanoparticles	20 mg/kg
3	Trastuzumab-dual loaded nanoparticles	10 mg/kg
4	Neratinib/Docetaxel nanoparticles	30 mg/kg
5	Neratinib/Docetaxel nanoparticles	20 mg/kg
6	Neratinib/Docetaxel nanoparticles	10 mg/kg
7	Trastuzumab/neratinib/Docetaxel sol.	30 mg/kg
8	Trastuzumab/neratinib/Docetaxel sol.	20 mg/kg
9	Trastuzumab/neratinib/Docetaxel sol.	10 mg/kg

## Data Availability

The original contributions presented in this study are included in the article/Supplementary Materials. Further inquiries can be directed to the corresponding author(s).

## Supplementary Materials

The following supporting information can be downloaded at: https://www.mdpi.com/article/10.3390/pharmaceutics17101265/s1, Figures S1–S12.

## Abbreviations

The following abbreviations are used in this manuscript:

ADC	Antibody Drug Conjugates
ADCC	Antibody Dependent Cell Cytotoxicity
ANOVA	Analysis of Variance
CAR T	Chimeric Antigen Receptor T-cell therapy
CI	Combination Index
DTX	Docetaxel
HER2	Human Epidermal growth factor Receptor
IC50	Inhibitory Concentration
MBC	Metastatic Breast Cancer
NCI	National Cancer Institute
NP	Nanoparticles
NTB	Neratinib
NTB-DTX-NP	Neratinib-Docetaxel loaded Nanoparticle
PGLA	Polylactitide-co-glycolide
TRZ-NP	Trastuzumab conjugated Nanoparticle
TGI	Tumor Growth Inhibition
TKI	Tyrosine Kinase Inhibitor
TRZ	Trastuzumab
