# Supplementary material for: Development of Novel Neratinib and Docetaxel Core-Loaded and Trastuzumab Surface-Conjugated Nanoparticle for Treatment of HER-2 Positive Breast Cancer
Source: Pharmaceutics. 2025 Sep 26;17(10):1265. doi: 10.3390/pharmaceutics17101265 (PMC12567355; doi:10.3390/pharmaceutics17101265)
Supplement: Supplementary file 1 [file pharmaceutics-17-01265-s001.zip › pharmaceutics-3808641-supplementary.pdf]

Development of Novel Neratinib and Docetaxel Core-Loaded and Trastuzumab Surface-Conjugated Nanoparticle for Treatment of HER-2 Positive Breast Cancer

Victor Ejigah, Gantumur Battogtokh, Bharathi Mandala and Emmanuel O. Akala *

Center for Drug Research and Development, Department of Pharmaceutical Sciences, College of Pharmacy, Howard University, Washington, DC 20059, USA
* Correspondence: eakala@howard.edu

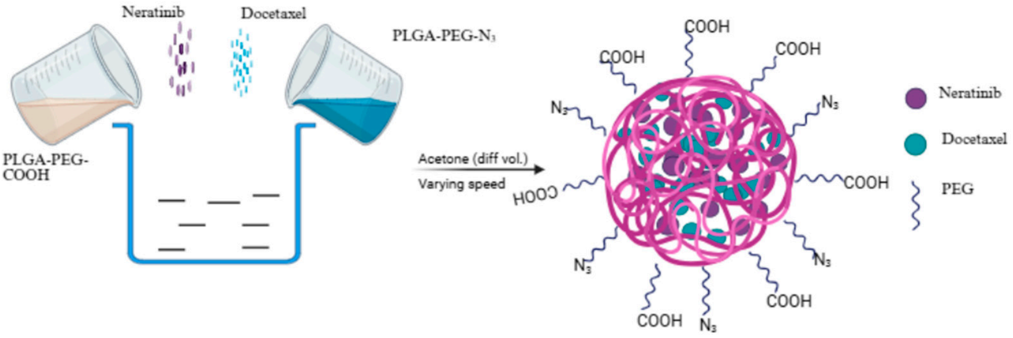

Supplement Figure S1: Formulation of dual-loaded neratinib and docetaxel nanoparticles. Blank and singly loaded neratinib and docetaxel nanoparticles were formulated likewise. Previous illustration removed and replaced with this one to better reflect nanoparticles

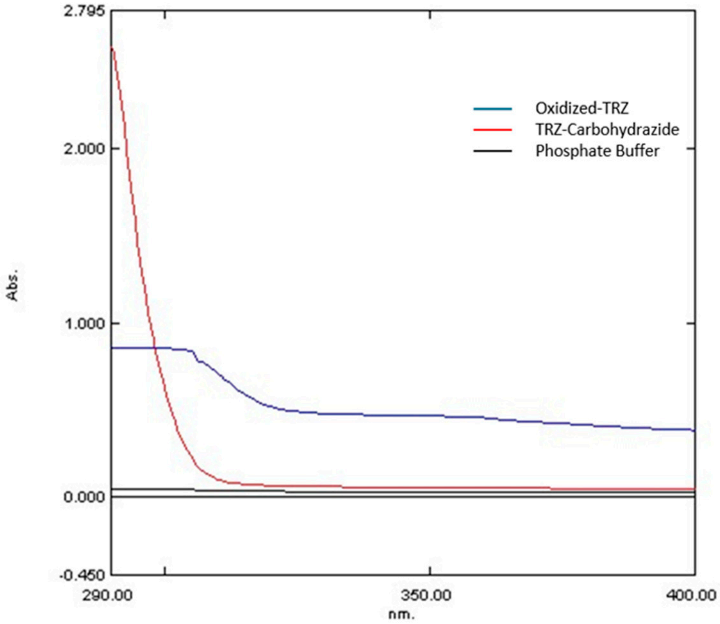

Supplement Figure S2: Relative absorbance of PBS (phosphate buffered saline), oxidized trastuzumab (Oxidized-TRZ) and trastuzumab-carbohydrazide conjugate PBS (0.045), oxidized trastuzumab (0.856) and trastuzumab-carbohydrazide conjugate (2.575).

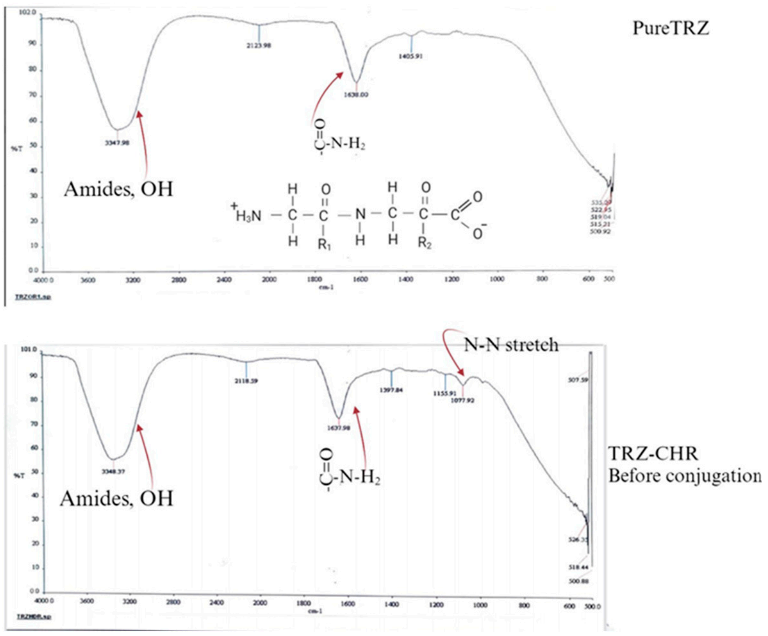

Supplement Figure S3: FTIR spectrum of trastuzumab before and after conjugation to carbohydrazide. The top image is the FTIR spectrum of trastuzumab before conjugation to carbohydrazide and the lower image is the spectrum after conjugation to carbohydrazide. TRZ-CHR: Trastuzumab-carbohydrazide

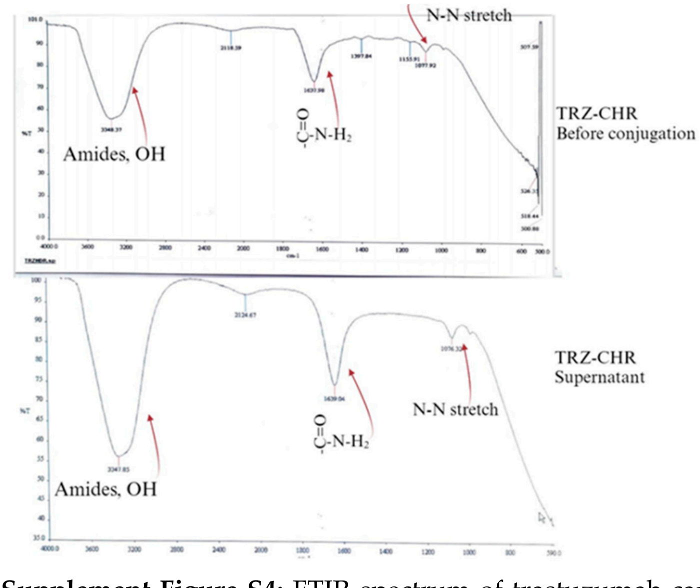

Supplement Figure S4: FTIR spectrum of trastuzumab-carbohydrazide before and after conjugation to nanoparticle

Top image: FTIR spectrum of trastuzumab-carbohydrazide before conjugation to nanoparticle; Lower image: FTIR spectrum of the supernatant after conjugation to nanoparticles

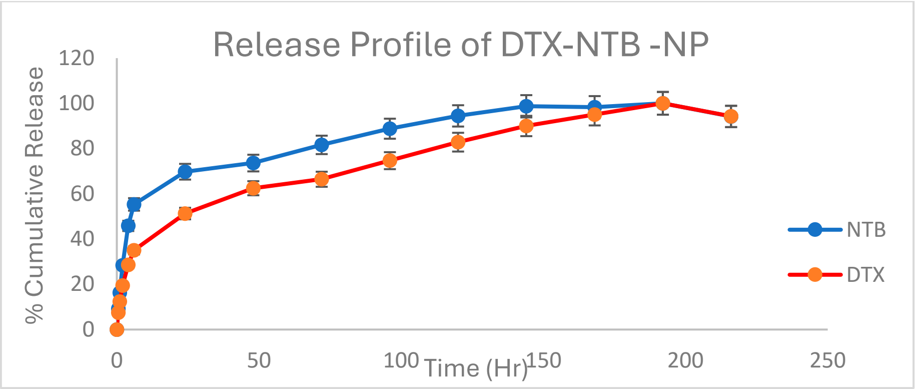

Supplement Figure S5a: Drug release profiles of analytes from NTB-DTX nanoparticles.

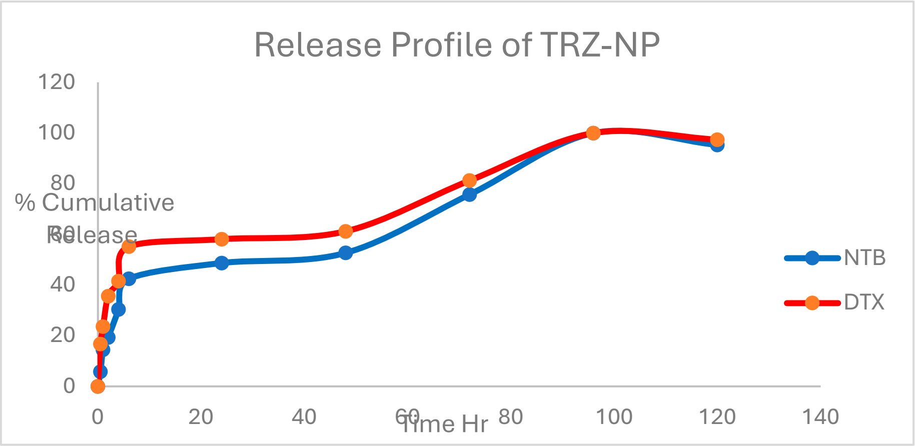

Supplement Figure S5b: Drug release profiles of analytes (neratinib &docetaxel) from TRZ- NP

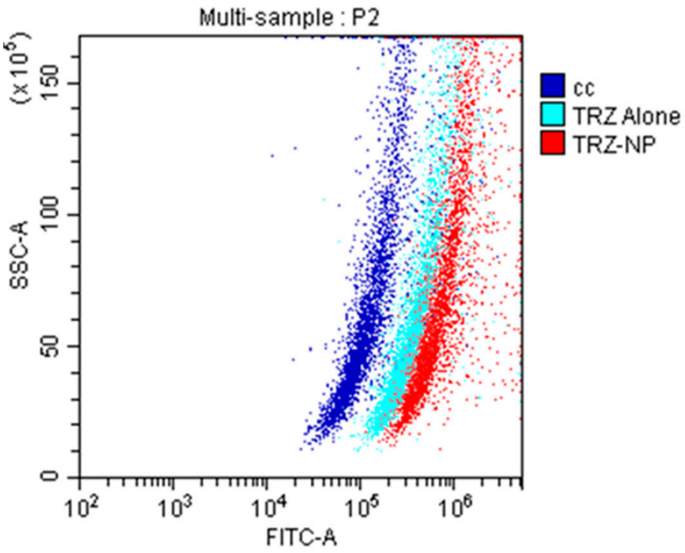

Supplement Figure S6a: Plot of SSC (side scatter) vs FITC cells positive cells for 3 treatment groups; TRZ, TRZ-NP and cc (HIgG1 control)

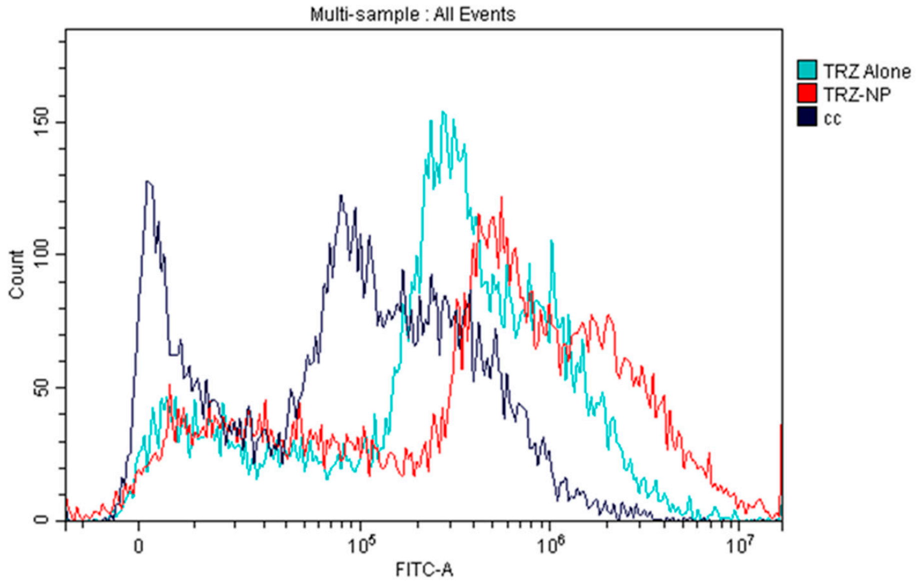

Supplement Figure S6b: Histogram overlay of the binding of Trastuzumab (TRZ), Trastuzumab-nanoparticle conjugate (TRZ-NP) and Human IgG1(HIgG1) with HER2+ over-expressing SKBR3 cells

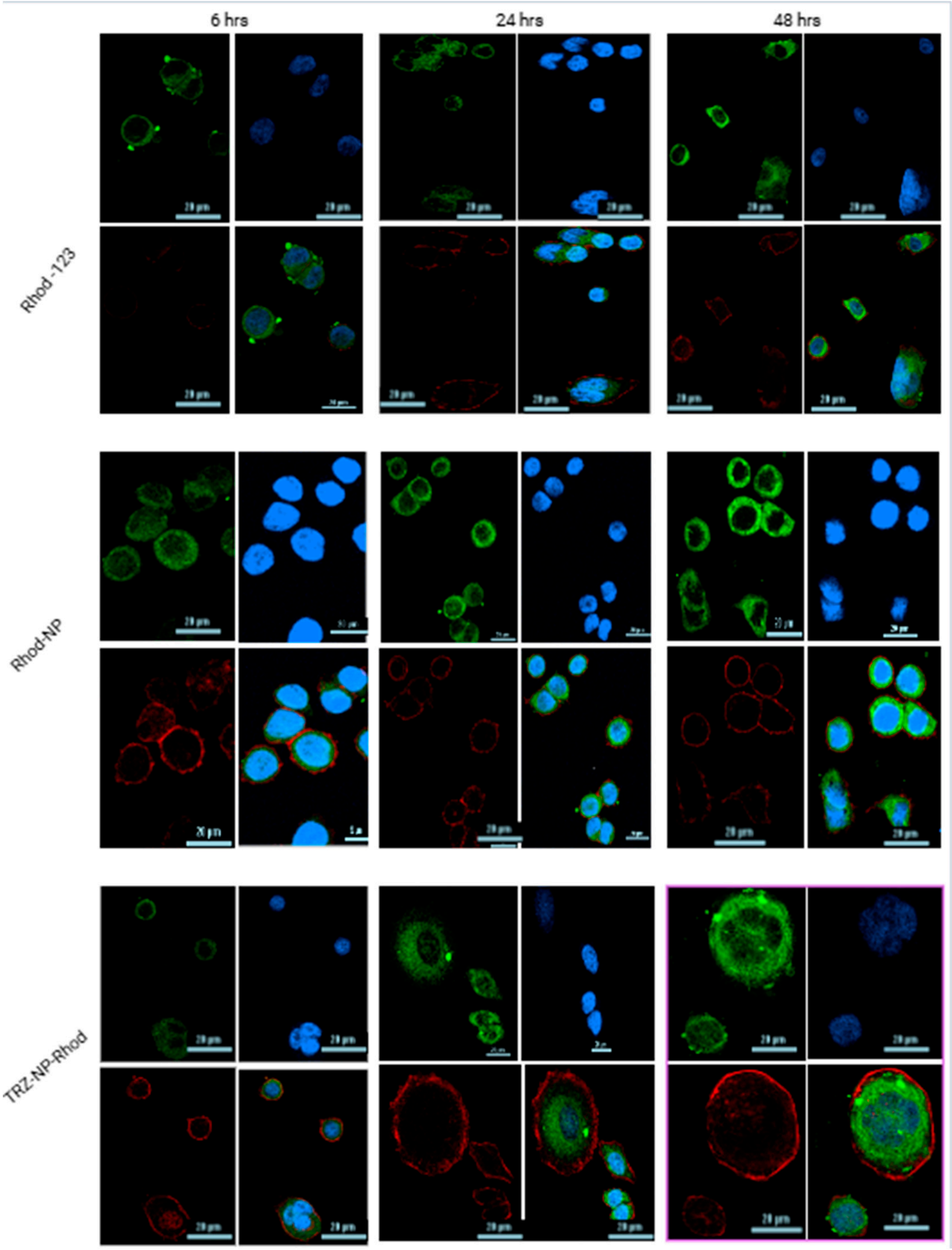

Supplement Figure S7: Internalization of nanoparticles by SKBR-3 cells at 6, 24 and 48 hours after incubation with fluorescent particles. Top left quadrant shows cell fluorescent nanoparticles only; top right quadrant shows nuclei staining only; bottom left quadrant shows cell membrane staining only; bottom right quadrant shows a merger of all quadrants.

TRZ-NP-Rhod: Rhodamine loaded nanoparticle conjugate; Rhod-NP: Rhodamine loaded nanoparticle; Rhod-123: Rhodamine-123

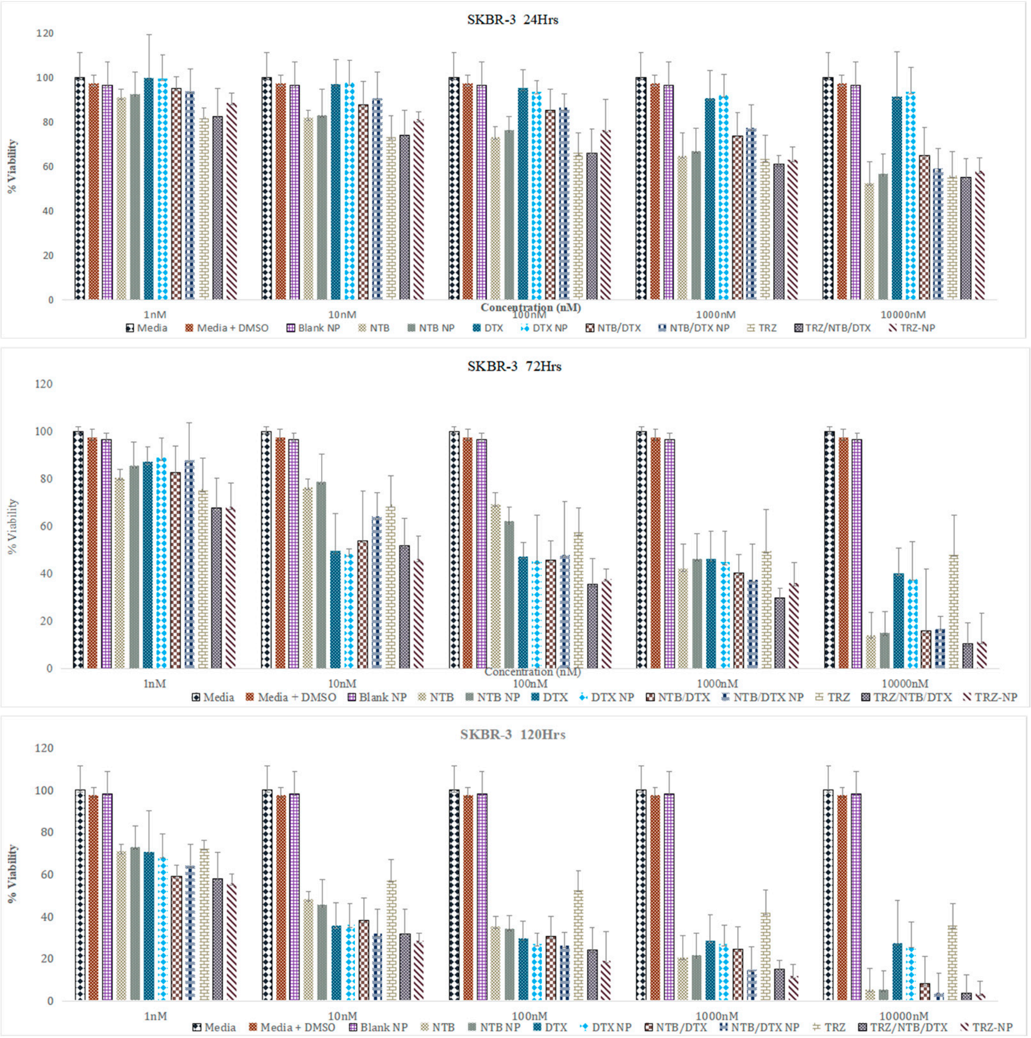

Supplement Figure S8: Percentage cell viability vs concentration plots for different concentrations of formulations.

NTB: Neratinib solution, DTX: docetaxel solution, NTB/DTX: neratinib/docetaxel solution, NTB-NP: neratinib nanoparticles, DTX-NP: docetaxel nanoparticles, NTB-DTX-NP: dual neratinib/docetaxel nanoparticles, TRZ: trastuzumab solution, TRZ/NTB/DTX: trastuzumab/neratinib/docetaxel solution and TRZ-NTB-DTX-NP: trastuzumab conjugated nanoparticles 24, 72 and 120hrs.

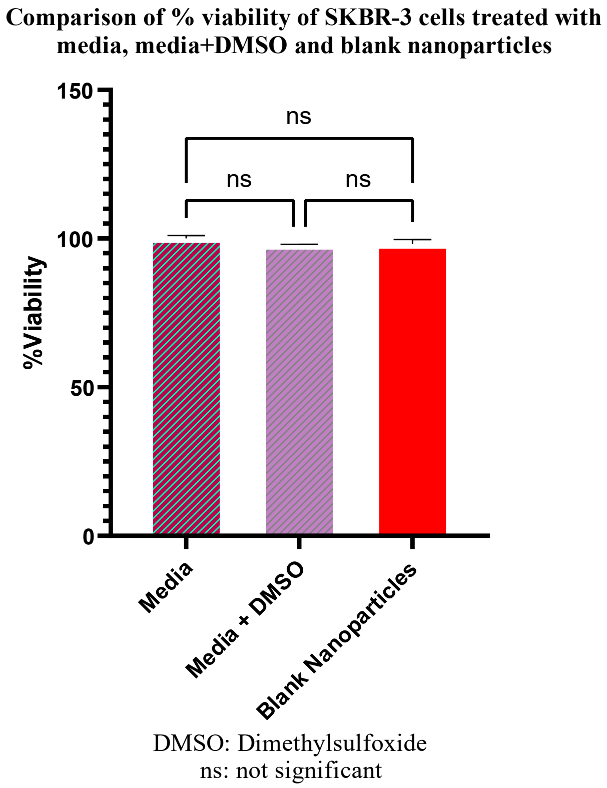

Supplement Figure S9: Plot of the effect of medium, medium + DMSO, and blank nanoparticles on % viability of SKBR-3 cells

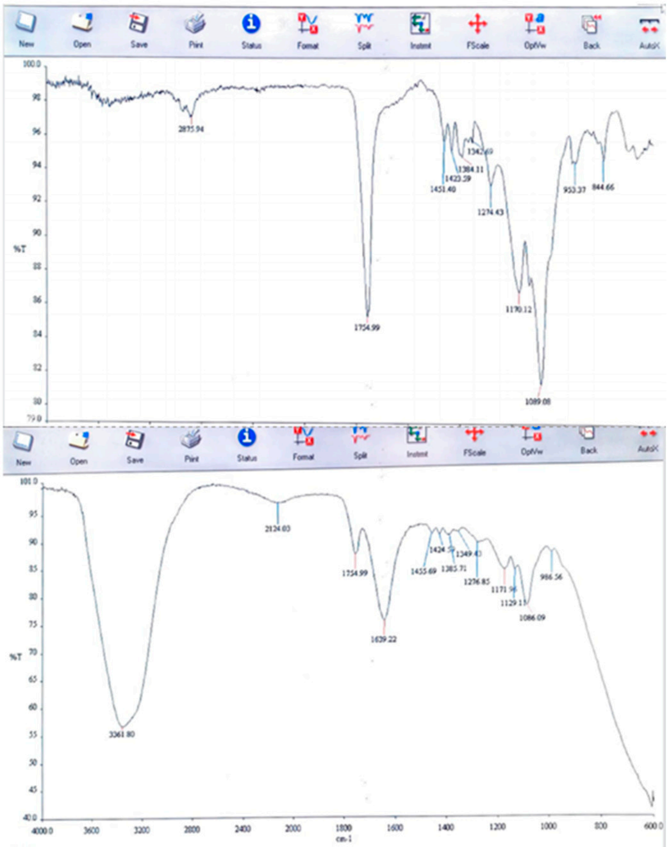

Supplement Figure S10: FTIR spectrum of blank nanoparticles and trastuzumab-conjugated nanoparticles. Top image: FTIR spectrum of blank nanoparticles; Lower image: FTIR spectrum of trastuzumab-conjugated nanoparticles

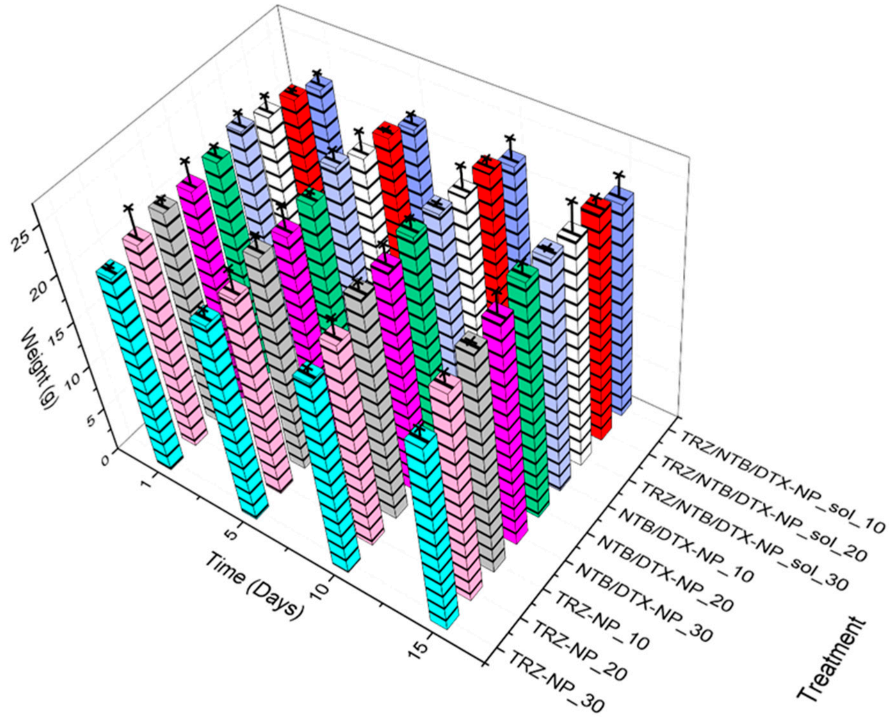

Supplement Figure S11: 3D plot of weight measures of mice exposed to escalating doses of different formulations. Weight was measured at four different time points. DTX: Docetaxel solution, DTX-NP: Docetaxel nanoparticle, NTB: Neratinib solution, NTB NP: Neratinib nanoparticles, NTB/DTX: solution, NTB/DTX-NP: Neratinib/docetaxel nanoparticle, TRZ: Trastuzumab, TND: Trastuzumab/Neratinib/docetaxel solution, TRZ-NP: Trastuzumab/Neratinib/Docetaxel conjugated nanoparticles.

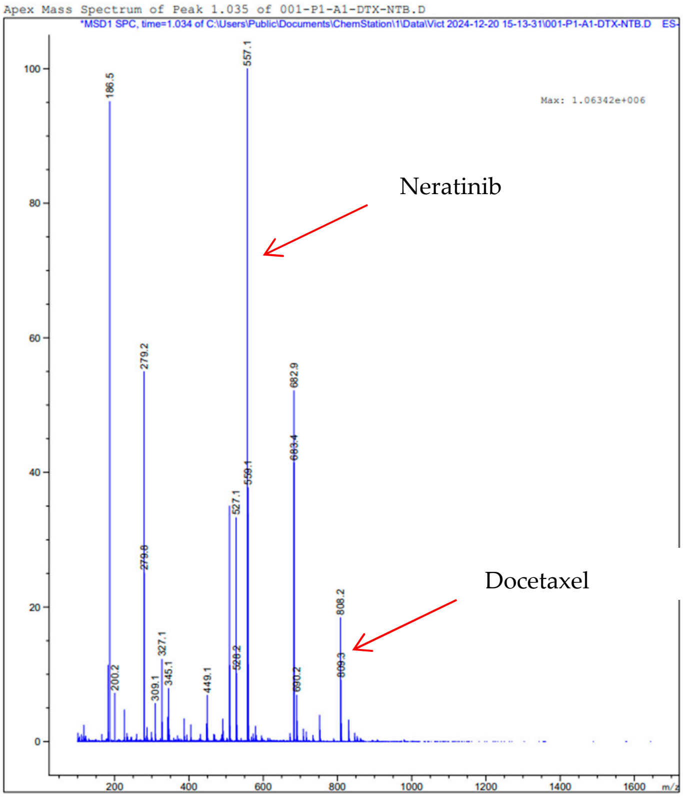

Supplement Figure S12: LC-MS spectrum from the 8th-day in vitro release sample of the dual-loaded nanoparticle shows neratinib with a mass of m/z 557.3033 and docetaxel with m/z 808.5367.
